# Clinical thyroidology: beyond the 1970s’ TSH-T4 Paradigm

**DOI:** 10.3389/fendo.2025.1529791

**Published:** 2025-06-24

**Authors:** Henry H. Lindner

**Affiliations:** Private Practice, Tunkhannock, PA, United States

**Keywords:** clinical medicine, deiodinases, guidelines, hypocortisolism, hypothyroidism, paradigm, reference range, T4/T3 combination therapy

## Abstract

The 2012 American endocrine associations' guidelines on hypothyroidism were a reiteration of the TSH-T4 Paradigm from the 1970s. They likewise defined hypothyroidism as hypothyroxinemia, assumed that almost all hypothyroidism was primary, and relied upon the thyroid stimulating hormone (TSH) test and inactive prohormone thyroxine (T4) for diagnosis and treatment. The guidelines’ authors acknowledged many TSH and other “pitfalls” in the paradigm yet warned physicians against attending to patients’ signs and symptoms and relative free T4 (FT4) and free triiodothyronine (FT3) levels—the only means by which to identify and avoid all pitfalls and provide individualized diagnosis and treatment. This inadequate paradigm has distorted medical practice and research for 50 years, including laboratories’ FT4 and FT3 reference ranges. It produces overdiagnosis, underdiagnosis, inadequate treatment, and widespread patient dissatisfaction. Since the 1970s, our understanding of thyroid hormone production, transport, metabolism, reception, and signaling has increased greatly, as has our appreciation of the importance of optimal T3 effects for health and wellbeing. Hypothyroidism must be defined physiologically as insufficient T3 effect in some or all tissues. The best indicators of tissue T3 effect are the patient’s signs and symptoms, and the best serum tests are FT4 and FT3, considered together. The TSH level is not a reliable indicator of T3 status in the untreated state and is oversuppressed by the peak levels that occur with once-daily oral T4 and/or T3. Normalizing an elevated TSH or low FT4 with T4 usually does not produce sufficient, let alone optimal, T3 effect and can leave some patients markedly hypothyroid. T4/T3 combination therapy is more physiological and effective than T4 monotherapy and must be guided by clinical criteria, not the TSH. Some patients cannot tolerate more T3 effect due to hypocortisolism, inflammation, and other disorders. There is no substitute for the practice of fully informed clinical medicine.

*The ultimate test of whether a patient is experiencing the effects of too much or too little thyroid hormone is not a measurement of hormone concentration in the blood or of the size and functional activity of the thyroid but of the effect of thyroid hormones on the peripheral tissues. Unfortunately, no simple, reproducible, and* sp*ecific tests are available to do this* (1).

## 1 Introduction

The biologically active human thyroid hormone is triiodothyronine (T3). It stimulates mitochondrial function and biogenesis (2, 3), gluconeogenesis (4), and glucose transport into cells, thereby increasing the energy available to every cell, tissue, and organ and improving their function. It also alters the expression of thousands of genes (5, 6). Optimal T3 effect is essential to every aspect of human development, health, and vitality, including the function of the rest of the endocrine system.

Under the influence of thyroid-stimulating hormone (TSH), secreted by the hypothalamic-pituitary (HP) system, the thyroid gland secretes thyroxine (T4) and T3. T4 is its dominant product but is only a prohormone—inactive until converted into T3. While the serum T4 level is largely determined by the HP system, the serum and intracellular T3 levels are largely determined by the intracellular deiodinases: D1, D2, and D3. Their amounts and activities vary from tissue to tissue. They locally increase or decrease T3 availability in a time-variable fashion (7, 8). Much of the T3 in the serum is derived from peripheral T4-to-T3 conversion (9). The serum FT4 level is three times greater than the FT3 level (in pmol/l). Different tissues obtain T3 by different mechanisms. Some preferentially take in T4 from the serum and convert it to T3 (e.g., brain, pituitary, heart, brown adipose tissue), whereas others are more reliant on serum T3 (e.g., liver, kidneys, adrenal glands) (10). We are still learning to appreciate the complexities and fallibilities of T3 transport, reception, and signaling (11). Therefore, the amount of T3 within the cells of any given tissue and the resultant T3 effect are dependent upon a complex chain of events, any link in which may be defective in any person, resulting in hypothyroidism in some or all tissues (12) (**Table 1**).

**Table 1:** Steps required to produce T3 effect.

HP production of a sufficient amount of active TSH
Thyroid gland response to TSH, producing sufficient T4 and T3
Active T4 and T3 transport across cell membranes
Intracellular T4-to-T3 conversion vs. T4/T3 deactivation
Rapid T3 effects on mitochondria and other cellular processes
Binding of T3 to cytoplasmic thyroid receptors
Interactions of T3-receptor complexes with co-regulators
Translocation of T3-receptor complexes to the nucleus
Binding of T3-receptor complexes to DNA response elements
Production of gene-transcription-related proteins and their effects

Hypothyroidism is the clinical state resulting from insufficient T3 effect in some or all tissues of the body. It varies from mild to severe and can variably affect different tissues. Its clinical presentation varies with the person’s genetics, cortisol status, medical conditions, nutrition, and inflammation. Hypothyroidism causes a vast array of signs and symptoms and causes or contributes to a host of medical and psychiatric disorders and diseases. (See **Tables 2**, **3** for partial lists.) Indeed, nearly every functional complaint and disorder encountered in medical practice can either be caused by hypothyroidism in whole or in part or be affected by it. Therefore, physicians must know how to determine every patient’s thyroid status and how to effectively diagnose and treat every variety and degree of hypothyroidism.

**Table 2:** Hypothyroidism: signs and symptoms.

Fatigue, excessive need for sleep	Lower body temperature
Cold intolerance, cold extremities	Muscle cramps, weakness, achiness
Myxedema: periorbital, ankle	Weight gain
Hoarse voice	Dry coarse hair and/or hair loss
Constipation, post-prandial bloating	Heartburn
Cognitive dysfunction (“brain fog”)	Dry, itchy skin
Headaches, often upon awakening	Depression and/or anxiety
Nasal stuffiness	Frequent infections
Numbness and tingling in extremities	Insomnia
Sleep apnea	Slow tendon relaxation

**Table 3:** Hypothyroidism: associated medical conditions.

Chronic fatigue syndrome	Fibromyalgia
Depression	Attention-deficit disorder
Hypercholesterolemia	Congestive heart failure
Atherosclerosis	Hypertension
Raynaud’s phenomenon	Carpal tunnel syndrome
Polycystic ovary syndrome	Polyneuropathy
Complications of pregnancy	Menorrhagia, amenorrhea
Infertility	Obesity
Non-alcoholic fatty liver disease	Gastrointestinal reflux

## 2 The TSH-T4 Paradigm

How does a physician decide if a patient is suffering from insufficient T3 effect? How does a physician intervene to optimize T3 effect in all tissues while avoiding overdosing? For answers, physicians look to guidelines published by endocrine associations. In their most recent guidelines, the American Thyroid Association (ATA) and the American Association of Clinical Endocrinology (AACE) claim to produce “evidence-based clinical guidelines for the clinical management of hypothyroidism” (13). However, their guidelines are neither clinical nor evidence-based. Their assumptions, analyses, and recommendations are all products of an approach invented in the 1970s that I call the “TSH-T4 Paradigm”. A paradigm is a set of ideas (e.g., definitions and assumptions) according to which evidence is gathered and interpreted. Contradictory evidence is often ignored or recategorized—as in this case. False paradigms can dominate sciences for decades or even centuries before they are replaced (14).

### 2.1 Clinical thyroidology before the TSH-T4 Paradigm

In the late 19^th^ and early 20^th^ centuries, physicians linked the conditions of myxedema and cretinism to the thyroid gland—to its damage or disease. They discovered that they could correct hypothyroidism with extracts of mammalian thyroid glands. Desiccated thyroid extract (DTE) was initially obtained from the thyroid glands of sheep, cows, or pigs. It was standardized by organic iodine content and contained T4 and T3 in ratios ranging from 2:1 to 5:1 (15). By the 1930s, most DTE was branded porcine thyroid, Armour Thyroid^®^ and Westhroid^®^, which had a reliable content and 4:1 T4/T3 ratio. DTE was the standard treatment for hypothyroidism from the 1890s to the 1970s. Physicians adjusted the DTE dose by clinical criteria—signs and symptoms. Experts affirmed the efficacy and safety of clinically adjusted DTE therapy (16). Although levothyroxine (T4) became available in 1955, most physicians continued to prescribe DTE because it was effective and normalized the serum protein-bound iodine (PBI). T4 monotherapy produced a high PBI and physicians feared that it would cause T3 deficiency. Restoring clinical euthyroidism required 2 to 3 grains (120 to 180mgs) of DTE, 200 to 400mcgs of T4, or 75 to 125mcgs of T3 (15, 17). T4/T3 ratios of 3.3:1 and 4:1 worked best to produce clinical euthyroidism and a normal PBI (15, 18).

### 2.2 Invention of the TSH-T4 Paradigm

In the early 1970s, a reliable test for TSH became widely available. Physicians discovered that most patients who were on clinically optimized DTE or T4 doses had low or suppressed TSH levels. They assumed that this indicated overtreatment, despite the lack of clinical evidence (19). Normalizing an elevated TSH required much lower DTE or T4 doses than recommended in the textbooks, often just one-half of the clinical dose (19–22). Research revealed that T4 was converted into T3 (23), alleviating the fear of T3 deficiency with T4 monotherapy. T4 produced more stable T4 and T3 levels, but higher FT4 and lower FT3 levels than seen in healthy persons.

A few endocrinologists believed that they could replace clinical diagnosis and DTE-T4/T3 treatment with a simple laboratory-based approach to diagnosis and treatment using T4 monotherapy to normalize the TSH: the TSH-T4 Paradigm (24, 25). They assumed that almost all hypothyroidism was primary. They cited studies that found that T4 doses that produced a low or suppressed TSH level and/or high FT4 levels were often associated with changes seen in hyperthyroidism, including increases in liver enzymes (26, 27), bone loss in women (28), and reduced cardiac systolic time intervals (29). An influential review concluded that “overzealous treatment” was producing “subclinical hyperthyroidism”, and that the goal of treatment should be a normal TSH (30). Some cited reports of thyrotoxicity with excessive doses of DTE (31) and T4/T3 (32), and the high T3 levels after doses to argue that DTE and T3 should not be prescribed.

From the start, it was clear that TSH-normalizing T4 therapy (TSHT4Rx) produced suboptimal clinical results. It produced Billewicz hypothyroidism scores (33) in the equivocal hypothyroid or, at best, the low euthyroid range (34). Adding 50mcgs of T4 to the TSH-normalizing dose raised Billewicz scores into the low euthyroid range and improved self-reported wellbeing (35) but was believed to be overtreatment. The paradigm’s architects ignored a T4 dosing study that found the TSH to be the least reliable, and the FT3 the most reliable serum indicator of clinical euthyroidism (36).

### 2.3 ATA/AACE’s TSH-T4 guidelines

The authors of the 2012 guidelines adhere to the TSH-T4 Paradigm but are conflicted. They similarly define hypothyroidism as an underactive thyroid gland indicated by a high TSH and/or low FT4. They state that the first goal of treatment is to resolve symptoms and admit that their guidelines cannot replace clinical judgment. However, they then warn physicians against attending to clinical criteria and using clinical judgment. After describing the euthyroid state as the “normalization of a variety of clinical and metabolic end points”, they thereafter equate euthyroidism with normal TSH and FT4 levels. They claim that neither clinical criteria nor relative FT4 and FT3 levels can be used to diagnose hypothyroidism, and “do not have sufficient specificity to serve as therapeutic endpoints…”. They provide no evidence or arguments for these beliefs; they are only assumptions of the paradigm (**Table 4**). They thus pay lip service to clinical thyroidology while promoting TSH normalization with T4 as the “standard treatment”.

**Table 4:** The TSH-T4 Paradigm’s assumptions.

TSH production and action are perfect, absent obvious HP disease.
TSH is a reliable inverse indicator of T3 effect in all tissues
Euthyroidism is a normal TSH level, absent obvious HP disease.
Almost all hypothyroidism is primary, evidenced by a high TSH.
Laboratories’ FT4 reference ranges also define “euthyroidism”.
The goal of oral T4 and/or T3 therapy is to normalize the TSH level.
T4-to-T3 conversion is always perfect—only T4 therapy is required.
T3 transport, reception, and signaling work perfectly in all persons.

The authors also assume that almost all hypothyroidism is primary—thyroid gland disease. They provide no evidence for this assumption because none exists. Such a study would require a definition of hypothyroidism that is not TSH-dependent and includes clinical and physiological criteria, FT4 and FT3 levels, and clinical response to a trial of effective T4/T3 treatment. (See below.) The authors frequently omit the adjective “primary” from “hypothyroidism”, thus reinforcing the idea that all hypothyroidism is thyroid gland disease evidenced by a high TSH, and thyroidology is just “TSH control” (37).

The authors consider central hypothyroidism (suboptimal TSH production) to be extremely rare, limited to cases of obvious HP disease or damage (~1 in 100,000 persons). They thus assume that every anatomically intact HP system functions perfectly. They neither state this assumption nor present any evidence to support it because it is false. (See TSH “pitfalls” below.) To this, the authors add the highly improbable assumption that the HP system responds to once-daily oral thyroid replacement therapy (TRT) exactly as it does to continuous endogenous thyroidal T4/T3 output. This too is contradicted by the evidence. They assert that a normal TSH level during the treatment of primary hypothyroidism with T4 is “euthyroidism”. To support this claim, they cite one old, small observational study (38), while almost all other studies refute it. (See below.)

The authors’ reliance on the TSH and T4 monotherapy requires the assumption that T4-to-T3 conversion is always perfect—sufficient to restore optimal FT3 levels and effects in all tissues if the FT4 is normal. They cite only one study to support this claim (39); while almost all other studies refute it. (See below.) Assuming perfect conversion, they see no reason to check a patient’s T3 level or prescribe T3. They state that a low FT3 should be ignored, as should persisting signs and symptoms of hypothyroidism, as long as the TSH and/or FT4 are normal. The authors discuss thyroid resistance separately because it is outside the paradigm, even though it is a cause of hypothyroidism as properly defined.

### 2.4 Acknowledged and unacknowledged TSH “pitfalls”

Reliance on the TSH test is illogical, as proven by the fact that the guidelines’ authors must warn physicians of 14 pitfalls to be avoided when attempting to rely on the TSH and its reference range (**Table 5**). Logically then, the TSH level is neither a sensitive nor specific indicator of thyroid (T3) status: It is the wrong test. The authors assume that physicians will somehow recognize and avoid all these pitfalls. How? There is only one way: by attending to the true indicators of T3 availability and effect—the relative FT4 and FT3 levels and, most importantly, the patient’s signs and symptoms. That is how each of the pitfalls was discovered and it is the only way to recognize them now. I will demonstrate that doing so results in the detection of more pitfalls (**Table 6**).

**Table 5:** ATA/AACE-acknowledged TSH pitfalls.

TSH-secreting tumors	Short-term variations in TSH levels
Central hypothyroidism	Low TSH with some medications
Biologically inactive TSH	High TSH with aging
High TSH in adrenal insufficiency	High TSH in general thyroid resistance
Low TSH with glucocorticoid therapy	Low TSH in acute illness, anorexia
Recent TSH suppression	High TSH in recovery from illness
Low TSH in early pregnancy	Laboratory interference by antibodies

**Table 6:** Unacknowledged TSH pitfalls.

Normal TSH in partial central hypothyroidism without evident HP disease
Normal TSH in peripheral resistance (e.g., deiodinase and receptor variants)
Normal TSH with aging with lower FT3 levels
High, low, or undetectable TSH on clinically adjusted TRT

Because they know that the TSH can mislead, the guidelines’ authors state that hypothyroidism must be “biochemically confirmed” by a low FT4. Logically then, physicians should check the FT4 first to establish the diagnosis of hypothyroidism and then check the TSH to see if it is primary or central. However, they know that relying on a normal (within reference range) FT4 also has pitfalls. T4 is inactive and T4-to-T3 conversion may or may not be compensatory. T4/FT4 levels can be increased or decreased by various non-thyroidal causes (40). Patients can be clinically hypothyroid with a normal FT4, and merely normalizing a low FT4 with T4 therapy will usually be inadequate. So, they attempt to rely on the TSH level, despite its many pitfalls.

The TSH-T4 Paradigm still dominates the practice of medicine today. Most physicians follow the ATA/AACE’s primary message: The TSH level tells them everything they need to know about a patient’s thyroid status, untreated or treated. They typically order only a TSH, or TSH with reflex—the FT4 is checked only if the TSH is abnormal. If the TSH is elevated, physicians usually prescribe just enough levothyroxine to normalize it. If the TSH is low on TRT, they declare the patient “thyrotoxic” and reduce the dose, even though the patient is feeling well and has no signs or symptoms of hyperthyroidism. If the patient feels worse on the lower dose, they will not raise it. They do not check the FT3 level and typically refuse to prescribe DTE or any T3. For patients, the results of this anti-clinical thyroidology range from suboptimal to catastrophic.

### 2.5 False diagnostic scheme

Because the TSH-T4 Paradigm is arbitrary, illogical, and isolated from clinical reality, it necessarily produces diagnostic and therapeutic errors: underdiagnosis, overdiagnosis, unnecessary treatment, and undertreatment. It is immune to correction by its clinical failures. The paradigm implies, and physicians follow, a simplistic two-test diagnostic scheme for the diagnosis of hypo- and hyperthyroidism and determination of the cause (**Table 7**).

**Table 7:** The TSH-T4 Paradigm’s diagnostic scheme.

Diagnosis	Criteria
Primary hypothyroidism	High TSH, low FT4
Primary hyperthyroidism	Low TSH, high FT4
Central hypothyroidism	Low TSH, low FT4
Subclinical hypothyroidism	High TSH, normal FT4
Subclinical hyperthyroidism	Low TSH, normal FT4

Since this algorithm does not include signs, symptoms, or relative FT4 and FT3 levels, it guarantees errors. It even creates two pseudo-diagnoses. A patient with a high TSH and normal FT4 is labeled “subclinical hypothyroidism”. However, the normal FT4 and/or FT3 levels may be low in-range, midrange, or high in-range. The patient may be clinically hypothyroid, euthyroid, or hyperthyroid, or may suffer from hypocortisolism. This is the bulk of the 15% of older adults who are diagnosed with hypothyroidism and given T4 (41). The low T4 doses usually produce no clinical improvement (42, 43) and can reduce T3 production, levels, and effects. (See below.) The scheme fails to diagnose partial central hypothyroidism and partial peripheral thyroid resistance because the TSH and FT4 are usually normal.

### 2.6 Patient dissatisfaction and physician disagreement

Since its invention, the TSH-T4 Paradigm has produced dissatisfaction among patients. Many testify that their health and vitality were restored only when they were diagnosed and treated clinically—according to their symptoms, not their TSH level. Most state that T4/T3 combination therapy, usually DTE with its 4:1 ratio, works better than T4 monotherapy, and that the doses they require produce a low or suppressed TSH (44–49). Patients with residual symptoms on T4 prefer T4/T3 and DTE (50–53). Published surveys have found prominent patient dissatisfaction with TSHT4Rx and greater satisfaction with DTE (54, 55). Patients reported that on DTE they experienced improved weight management, energy, mood, and memory (56). Many experienced physicians, including some prominent former advocates of the paradigm, have argued that hypothyroidism should be treated according to clinical criteria, not the TSH, and/or that treatment should include T3 (57–69).

## 3 Why the TSH-T4 Paradigm fails to diagnose most hypothyroidism

### 3.1 Misunderstanding and misuse of endocrine reference ranges

Most endocrine tests are reported with broad population ranges, not with physician-adjudicated ranges. Their limits are just population statistics: two standard deviations (SDs) from the mean of the tested group. This includes the middle 95% of the tested population and so defines almost everyone as “normal”. Only the highest and lowest 2.5% are reported as “high” or “low”. To be used as diagnostic ranges, the persons tested to produce the ranges should be very carefully screened for any signs or symptoms of deficient or excessive hormone effect. They should also have ideal-optimal health and vitality. However, the subjects are not screened for symptoms; laboratory guidelines do not call for it (70). In addition, all subjects would need to have the same hormone sensitivity.

To produce a suggested reference range, a test kit manufacturer usually samples 120 or so “apparently healthy” persons. They are screened only for diseases and medications. The lack of symptom screening and the use of 2SDs to define “normal” explain the breadth of endocrine ranges. The upper limits are typically 2 to 5 times greater than the lower limits, and up to 30 times greater (**Table 8**). The ranges are far from representing “the most normal of normals”, as often claimed.

**Table 8:** Breadth of endocrine reference ranges.

Test	Range	Multiplier
Male free testosterone	35 to 155pg/ml	4.4x
Female free testosterone	0.2 to 6.4pg/ml	32x
Free T4	0.8 to 1.8ng/dl	2.3x
Free T3	2.0 to 4.4pg/ml	2.2x
AM serum cortisol	5 to 20mcg/dl	4x
AM saliva cortisol	0.02 to 0.6mcg/dl	30x
DHEA sulfate	30 to 300 mcg/dl	10x

A large problem in the interpretation of hormone levels is their greater degree of individuality compared to most of the tests that physicians perform every day—tests that have narrow ranges and low degrees of individuality (e.g., serum sodium levels). Physicians carry their training and experience with narrow-ranged, low-individuality tests over to the broad-ranged, high-individuality endocrine tests and so think that a hormone level anywhere within the laboratory’s range is “sufficient”. They will often ignore a hormone level that is “a little low”. However, a healthy person’s hormone levels vary much less with time than the breadth of the population range (high degree of individuality) (71). Hormone effects also exist on a continuum from lower to higher levels. Consider that patients could have FT4 and FT3 levels that are one-half of their previous levels yet still test as “normal”. Even if we had perfectly accurate tests and all persons had the same hormone sensitivity and milieu, the placement of dividing lines between “deficient”, “sufficient”, and “excessive” would still be an arbitrary act. Reference ranges only provide a statistical description of the population studied. They do not define what is optimal for the human species, let alone for any individual.

### 3.2 Distortion of FT4 and FT3 reference ranges

The FT4 and FT3 ranges reported by laboratories are distorted by a practice that is unique in laboratory science. As a result of the TSH-T4 Paradigm, laboratory scientists believe that a normal TSH equals euthyroidism. Therefore, they think it appropriate to include FT4 and FT3 levels from physician-ordered thyroid panels in their reference range determinations—if the TSH was normal. Their ranges thus include symptomatic untreated and T4-treated clinic and hospital patients. The result is evident in their ranges’ increased breadth compared to those found in studies of non-patients. With kits that yield similar values, laboratories report FT4 ranges with lower limits of only 0.6 to 0.8ng/dl and upper limits of 1.8 to 2.2ng/dl. The upper limits are 2 to 3 times greater than the lower limits. In contrast, studies of non-patient populations like blood donors and soldiers, without symptom screening, consistently yield narrower 2SD FT4 ranges of around 1.0 to 1.65ng/dl (72–78). The results of this misuse of the TSH test were documented in a test kit manufacturer’s detailed report (**Table 9**). The advised reference ranges included patient data (79). The large differences in the lower limits indicate that dysfunctional TSH secretion and T4-to-T3 conversion are much more common than primary hypothyroidism, which affects only 0.3 to 0.4% of the population (80, 81).

**Table 9:** Non-patient vs. patient ranges.

FT4: Non-patients	0.99 to 1.62ng/dl
FT4: Clinic/hospital patients	0.85 to 1.74ng/dl
FT3: Non-patients	2.5 to 4.3pg/ml
FT3: Clinic/hospital patients	2.04 to 4.4pg/ml

Laboratories using similar kits should report FT4s with a lower limit of 1.0ng/dl (range: 1.0 to 1.65) and FT3s with a lower limit of 2.5pg/ml (range: 2.5 to 4.3). However, these are still symptom-unscreened population ranges, and diagnosis should never be made by a laboratory test alone. FT4 and FT3 test results can also be inaccurate (82–84) and patients vary greatly in their need for, and response to T4 and T3 levels. There is no laboratory substitute for the physician’s clinical judgment.

### 3.3 FT4 and FT3 levels matter—within their reference ranges

The insensitivity of the TSH test and FT4/FT3 population ranges is illustrated by many published studies that associate indices of better well-being and health in untreated persons with higher compared to lower FT4 and/or FT3 levels within their ranges. For instance, higher T4 levels are associated with better cognitive function (85, 86) and lower risk of cognitive decline (87). Lower maternal FT4 levels in the first trimester of pregnancy are associated with lower neonatal neurobehavioral scales (88), impaired psychomotor development (89), and autism (90). Lower T4 and T3 levels are associated with depression or a worse prognosis for remission of depression (91–94). Persons with lower FT4 levels complain more of myalgias and weakness and have lower muscle strength on testing (95). Higher FT4 levels are associated with lower all-cause mortality and higher FT3 levels with lower cancer mortality (96). Higher FT4 levels are associated with less arterial stiffness, lower diastolic blood pressure, and less insulin resistance (97). Carotid artery intimal thickness is inversely associated with FT4 levels (98, 99). Persons undergoing cardiac catheterization who have FT3s in the highest tertile have half the incidence of severe coronary atherosclerosis as those in the lowest tertile (100). Lower FT4 levels are associated with hypercoagulability (101), higher body mass index and weight gain (102), subcutaneous fat (103), metabolic syndrome (104–106), and non-alcoholic fatty liver disease (107). Lower FT3 levels are also associated with a lower metabolic rate and weight gain (108). One can find studies that do not show these correlations or, in some cases, show the opposite. A sufficient explanation is that a FT4 or FT3 level by itself is insufficient to assess the thyroid status of any individual. They must be considered together to obtain the best serum estimate. (See below.)

### 3.4 Nature of the TSH-FT4 population correlation

**Figure 1:**
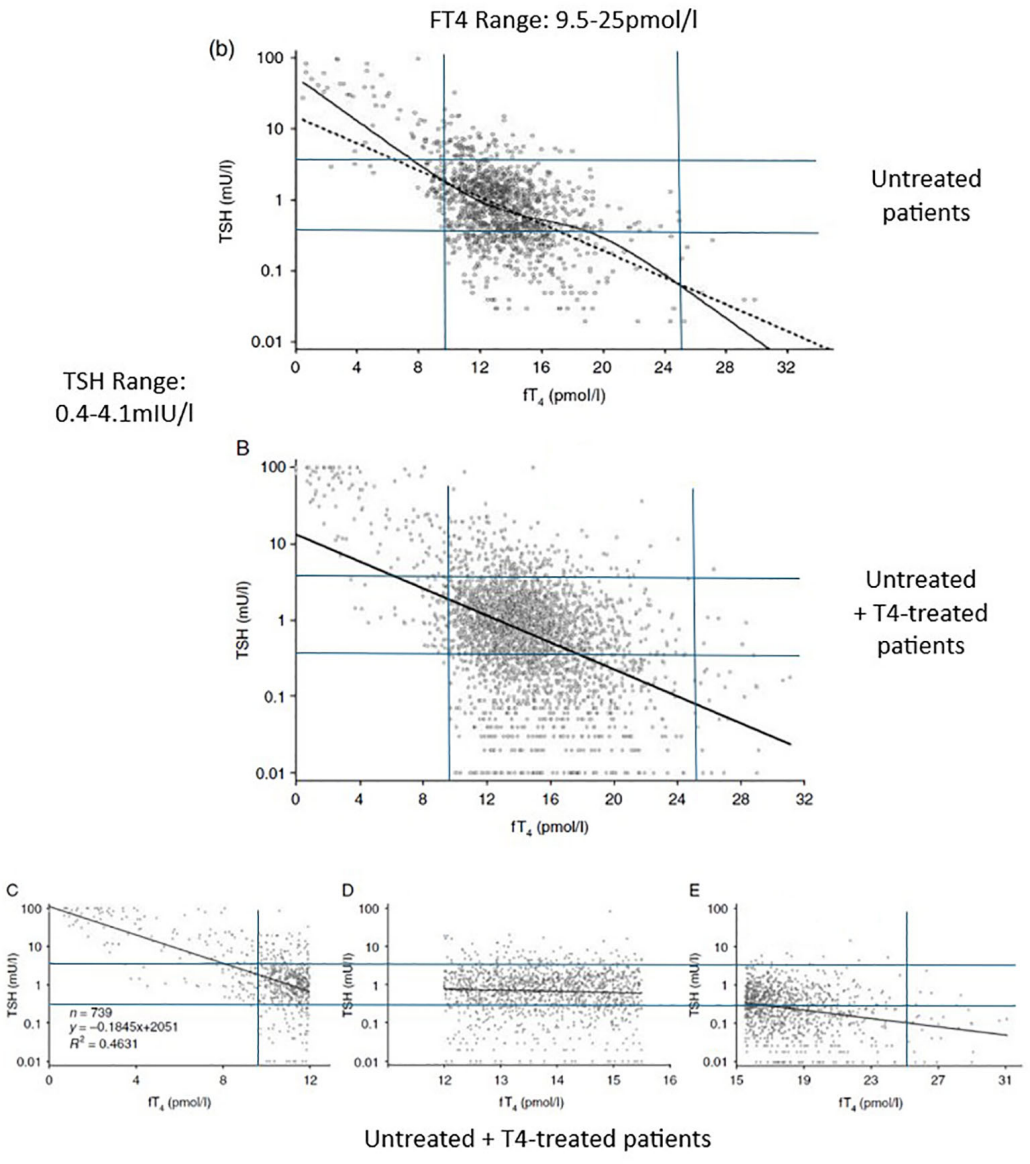
The TSH-FT4 correlation seen in untreated **(b)** and untreated plus T4-treated patients **(B–E)**. The TSH and FT4 reference ranges (in blue) were added by the author (by permission of Oxford University Press). Adapted with permission from ‘Complex relationship between free thyroxine and TSH in the regulation of thyroid function’ by Hoermann R, Eckl W, Hoermann C, Larisch R, license number 6043140018466, Oxford University Press.

The ATA/AACE’s emphasis on the TSH test encourages physicians to think that the higher the patient’s TSH level, the lower their thyroid (T3) status, and vice versa, even within the TSH range. This is an example of the ecological fallacy, i.e., drawing an inference about an individual from a correlation seen in the group (109). The best-fit lines in **Figure 1** show the general inverse correlation between TSH and FT4 levels in a population of untreated (b) and untreated plus T4-treated patients (B) referred to a tertiary center (110). Patients with HP disease/damage were excluded. Reliance on the TSH might be defensible if every data point were close to the best-fit line. However, the data points are widely scattered—very high and low TSH levels coexist with normal FT4 levels and vice versa. The scatter is greater in the T4-treated patients. The group correlation clearly has no relevance to any individual. The general group correlation is also absent in the middle of the FT4 reference range, where the slope is almost flat. The TSH-FT4 correlation has strength only at higher and lower FT4 levels due to the presence of a subgroup with the correlation: patients with primary hypothyroidism and hyperthyroidism. The large scatter in the TSH levels attests to the high degree of individuality in HP-thyroidal axis function. The TSH level is clearly an unreliable inverse indicator of any patient’s FT4 level.

### 3.5 Why TSH is not a reliable indicator of T3 levels or effects

The TSH’s many pitfalls arise from the attempt to use the level of a stimulatory pituitary hormone as a surrogate indicator of end-hormone levels and effects. It is for good reasons that physicians do not attempt to do so with the gonadal and adrenal systems. It is equivalent to claiming that one’s home heating thermostat is working perfectly even as the house continues to get colder. Absent disease of the primary gland, the HP system controls serum hormone levels, and it is neither perfect nor passive. The hypothalamus and pituitary gland are parts of the brain and are affected by inputs from many regions of the brain and by many neurotransmitters. The HP system is also part of the larger neuro-endocrine-immune system and is affected by genetic variants, cytokines, illnesses, nutritional factors, chemicals, drugs, stress, etc. Because the HP system is much more complex than the primary gland, it is much more likely to be dysfunctional. It should be so judged when the clinical and laboratory evidence so indicates.

TSH is produced and secreted as a mixture of isoforms, the majority of which have differences in their oligosaccharide structure and possess different bioactivity (111, 112). Dysfunctional central hypothyroidism has been associated with relatively inactive forms of TSH, genetic variants, and other molecular disorders (113–115). There are published reports of patients with central hypothyroidism who had normal HP imaging studies (116–119). Most patients with central hypothyroidism due to known HP damage/disease have normal TSH levels and often have normal, but low-in-range FT4 levels (120–122). This “euthyroid” central hypothyroidism is another pitfall in the paradigm. The HP-thyroidal system also becomes dysfunctional with age. Between the ages of 20 and 80, the TSH response to low FT4 levels declines by 75% (123).

The HP system necessarily responds differently to serum T4 and T3 levels than other tissues, in some persons pathologically so. Whereas other tissues have separate membrane transporters for T4, T3, and reverse T3 (rT3), the pituitary has one transporter for all three (124) and it is not energy-dependent (T3-dependent) as are those in other tissues (125, 126). There are also four different T3 receptors (TRs) with various tissue distributions. The HP system primarily expresses TRβ2 (127). The function of any of these tissue-specific proteins can be altered by single nucleotide polymorphisms (SNPs) in any individual.

In mild thyroid gland failure, the elevated TSH may be perfectly compensatory—maintaining T3 effect in the periphery by assuring sufficient thyroidal T4/T3 output and peripheral T4-to-T3 conversion. (See below.) Other persons with the same higher TSH may be hypothyroid. Some may be hypothyroid with a normal or low TSH due to dysfunctional TSH hyposecretion or partial peripheral thyroid resistance (e.g., reduced T4-to-T3 conversion), or both.

Physicians defend the paradigm’s reliance on the TSH test by touting its “sensitivity”. The TSH level does respond in a logarithmically amplified way to changes in the serum FT4 level in any given person. In addition, the latest-generation TSH test is highly precise; it can measure the TSH to within 1/1000^th^ of one mIU/l. However, neither of these facts implies diagnostic sensitivity or specificity. In fact, a TSH level alone implies nothing certain about any individual’s T3 status. It has no meaning apart from the FT4 and FT3 levels and the clinical context.

### 3.6 Why FT4 is not a reliable indicator of T3 levels or effects

The FT4 level indicates the serum availability of the more abundant prohormone. Compared to the TSH, it has a more reliable causal relationship with T3 availability and therefore T3 effect. The ATA/AACE argue that one can rely on the FT4 level because when “sufficient” T4 is available the deiodinases will adapt and assure optimal intracellular T3 availability and effect in every tissue. Some have proposed that the FT4 replace the TSH as the primary measure of thyroid status (128).

However, the FT4 level is still an indirect indicator. While the serum T4 level is controlled by the HP system, the production and elimination of T3 are largely controlled by intracellular deiodinases: D1, D2, and D3. Their concentrations and activities vary from tissue to tissue (8). They are affected not only by serum T4, T3, and TSH concentrations but also by many other factors: e.g., genetic variants, aging, inflammation, cytokines, nutrients, chemicals, cortisol, testosterone, growth hormone, etc.

In untreated primary hypothyroidism and less so in central hypothyroidism, deiodinases do attempt to compensate for the relative lack of T4 by increasing their conversion of T4 to T3. Patients with primary hypothyroidism are much less symptomatic if their FT3 is within range rather than low (129). When conversion is vigorous and compensatory, the FT3 can be above mid-range or even high. However, people with a mildly elevated TSH who lack compensatory conversion can have FT4 and FT3 levels that are “normal”, but if both are low in their ranges can suffer from hypothyroidism—even myxedema coma (130). T4-to-T3 conversion declines with aging. TSH and FT4 tend to rise while FT3 steadily declines, with deleterious effects (131).

The deiodinases are altered by genetic variants in many persons—another anomaly for the paradigm. A large percentage of the population has D1 and/or D2 variants that reduce T4-to-T3 conversion. The C allele of locus rs225014 of D2 (a.k.a. Thr92Ala) has a frequency of 39% in the population, so 15% of persons are homozygous. On T4 monotherapy post-thyroidectomy, homozygotes have lower FT3 levels (132). They have lower quality of life scores and experience significant improvement on T4/T3 combination therapy (133). The T allele of locus rs11206244 of D1 (a.k.a. C785T or D1a C/T) has a frequency of 34%, so 12% of the population is homozygous. Homozygotes have reduced T4-to-T3 conversion and reduced rT3 elimination. They have higher rT3/T4 ratios and lower T3/rT3 ratios (134). rT3 is not just inactive; it inhibits D2 activity and thereby T4-to-T3 conversion (135–138). Homozygotes have a greater tendency to major depression (139) that responds to T3 supplementation (140). Given these alleles’ frequencies, 13% of the population is heterozygous for both D1 and D2 variants, and 2% is homozygous for both.

Heterozygosity for either one of these variants has been associated with hypothyroid symptoms and various health problems, while having little effect on serum TSH, FT4, or FT3 levels (141). Homozygosity further impairs T4-to-T3 conversion. Homozygosity for one or even both of these known variants (i.e., partial peripheral hypothyroidism) is much more common than primary hypothyroidism. Many persons with deiodinase variants will be sufficiently symptomatic that they require T4/T3 therapy. A few require T3 monotherapy (142).

Other forms of genetic and acquired peripheral thyroid resistance are more common than currently acknowledged (143). Any one of the many steps involved in producing T3 effect (**Table 1**) can be defective in some or all tissues (144–146). Many of these proteins vary among the tissues, so peripheral thyroid resistance can be tissue-specific. There are at least 10 active T4 and T3 transport systems with variable tissue distributions (147, 148). There are four known T3 receptors and resistance syndromes have been associated with variants of each (149), some of which produce hypothyroidism while TSH, FT4 and FT3 levels remain normal (150, 151). There are at least 13 thyroid receptor-interacting proteins (TRIPs) that modulate T3 signaling and effect in various tissues (152). Each of these proteins’ functions can be affected by SNPs, nutritional deficiencies, toxins, and other factors. Each protein is also a possible target of autoimmunity. Unlike generalized thyroid resistance that also affects the HP system, in peripheral resistance the TSH is usually normal as are the FT4 and FT3 levels (134).

### 3.7 Most persons suffering from hypothyroidism have normal “thyroid function” tests

The preceding discussion explains why normal “thyroid function tests” (TSH and FT4) do not imply that a person has sufficient, let alone optimal T3 levels and effects in all tissues. Of course, population studies find that hypothyroid symptoms are more common in persons with low FT4 levels than in those with normal FT4 levels (153, 154). This is an expected population correlation; it does not justify defining hypothyroidism as a “low FT4”. What matters is T3-related physiology—of which symptoms are the most sensitive indicator. The ATA/AACE attempt to dismiss symptoms as guides to diagnosis by citing a study that found that hypothyroid symptoms were prevalent in persons with normal TSH and FT4 levels (81). However, if one defines hypothyroidism as insufficient T3 effect one draws a very different conclusion.

In that study of persons attending a health fair, of those who were not taking thyroid medication 8.5% had “subclinical hypothyroidism” and only 0.4% had “overt hypothyroidism”. However, “euthyroid” (TSH-T4 normal) persons were nearly as likely to have hypothyroid signs and symptoms as “subclinical hypothyroid” persons. Using the Billewicz score, “euthyroid” persons were also nearly as likely as “overt hypothyroid” persons to have moderate hypothyroidism (48% vs. 52%). Among those with Billewicz scores representing severe hypothyroidism, “euthyroid” persons outnumbered “overt hypothyroid” persons by a 50-to-1 margin (20% of 22,842 vs. 80% of 114).

The ATA/AACE guidelines’ authors dismiss the possibility of hypothyroidism in symptomatic persons with normal TSH and FT4/FT3 levels by citing one study in which such patients and healthy controls were given 100mcgs of T4 daily or placebo (155). T4 treatment caused both groups to have lower SF-36 quality of life scores, while scores improved on placebo. This subreplacement T4 dose lowered TSH and raised FT4 and FT3 levels. The timing of the blood test relative to the last dose was not stated. The FT3 rise was far less in the patient group, suggesting reduced T4-to-T3 conversion. The poor clinical result suggests that this T4 dose reduced T3 effect in some tissues. It was not an effective trial of thyroid optimization (See below). In contrast, when patients with normal TSH and FT4 levels but long-standing hypothyroid symptoms were treated with up to 200mcgs of T4 according to clinical criteria, most benefitted dramatically (156). In a case series of “euthyroid” patients, T3 and T4 prescribed to optimize the T3/rT3 ratio and maintain a low-normal FT4 produced dramatic improvements in symptoms and many disorders (66). I have seen similar dramatic improvements with prescribing T4/T3 therapy to symptomatic “euthyroid” persons. Most had low-in-range FT4 and/or FT3 levels but a few had mid-range levels.

## 4 Why the TSH-T4 Paradigm fails to produce euthyroidism

Consider that even if TSH production and T4-to-T3 conversion were perfectly vigorous in a patient, the many checks and balances in the complex thyroidal system make it highly improbable that an unphysiological intervention like once-daily oral T4 or even T4/T3 therapy could produce optimal tissue T3 levels and effects in all tissues while producing the same serum TSH, FT4, and FT3 levels seen in euthyroid controls. In fact, there is only one way to do so. In a landmark study involving thyroidectomized rats, investigators measured the TSH and the serum and tissue levels of T4 and T3 in animals given various amounts of T4 and/or T3 by continuous infusion. A T3 infusion that produced normal serum T3 levels failed to restore T3 levels in many tissues (157, 158), as expected in the absence of T4. A T4 infusion failed to restore T3 levels in the serum and some tissues until T4 levels were supraphysiological and the TSH suppressed (159). Only a continuous infusion of T4 and T3 in a 6:1 ratio restored both serum and tissue levels of T4 and T3 to those of controls without suppressing the TSH (10). In another study of thyroidectomized rats, normalizing the TSH with T4 delivered continuously by subcutaneous pellets also failed to restore T3 levels in the serum and some tissues; to do so required the addition of T3 (160).

### 4.1 HP system is uniquely sensitive to T4

The inability of T4 monotherapy, even by continuous infusion, to restore serum TSH, T4, and T3 levels and T3 levels in many tissues to those of controls can be explained. To start, the thyroid gland secretes T3 in addition to T4. Also, as the thyroid controller, the HP system cannot attempt to maintain stable intracellular T3 levels in the face of changing serum FT4 and FT3 levels. It must react directly and proportionally to changes in FT4 and FT3, with more or less TSH production, in order to stabilize those levels. Since T4 is the dominant product of the thyroid gland, it is understandable that the HP system is especially sensitive to serum T4. In the hypothalamus, D2 is not ubiquitinated and thereby not deactivated by its interaction with T4 as in other tissues. As T4 levels rise, the HP system maintains its D2 expression and activity and thereby its T4-to-T3 conversion (161, 162) to reduce TSH secretion and avoid thyrotoxicosis (163). Therefore, on T4 monotherapy the degree of TSH suppression is disproportionate to the amount of T3 effect in other tissues (160). The TSH level only reflects the T3 effect in the HP system.

### 4.2 Oral T4 and/or T3 peak levels over-suppress the TSH for 24 hours

Unlike the rat studies mentioned above, we administer TRT by mouth, and usually once daily. This dumps the entire day’s T4 or T4/T3 into the circulation within a few hours, producing unphysiological peak levels. Four hours after a daily T4 dose, FT4 levels are 13% to 36% higher, and FT3 levels 8% higher than at the 24-hr trough (164–166). Three hours after a large daily T3 dose, FT3 levels are 100 to 300% higher than at the 24-hr trough. Even with these FT4 and FT3 peaks and troughs, the TSH level varies little over 24hrs (167). A single large T3 dose (50mcgs) given to controls reduced the TSH to a nadir of 0.5mIU/l at 12hrs. At 24hrs, the TSH was still near its nadir (0.55mIU/l) even though the FT3 had nearly returned to baseline (168). In rats, a rapid T4 infusion suppressed TSH production for over 22 hours (169). The over-suppression of the TSH by once-daily T4 therapy in patients can also be seen in **Figure 1**. by comparing graph (b) (untreated) to graph B (untreated and treated patients).

If the peak levels are reduced by splitting the daily dose, the TSH is less suppressed. A medical statistician determined that each additional splitting of his daily T4/T3 dose increased his TSH by ~1.0mIU/l (**Supplementary 1**). Compared to adding 6mcgs of immediate-release T3 to patients’ daily T4 dose, adding 6mcgs of slow-release T3 produced lower peak T3 levels and higher trough TSH levels (170). Since the peak levels after once-daily oral TRT oversuppress the TSH, neither the population TSH reference range nor the patient’s own premorbid TSH level can be the goal of therapy. The treated TSH will usually need to be lower than the non-treated TSH.

### 4.3 TSH- and FT4-normalizing T4 therapies fail to restore T3 levels and effects

When there is thyroid gland dysfunction, the higher TSH is compensatory, stimulating additional thyroidal T4/T3 production and additional D1- and D2-mediated T4-to-T3 conversion not only in the thyroid gland but in many non-thyroidal tissues as well (171–173). In both thyroidectomized dogs and humans on T4 replacement therapy, TSH injections raised serum T3 levels by approximately 50% and lowered rT3 levels (174, 175). Higher serum T3 levels stimulate D1-related T4-to-T3 conversion and degradation of rT3 (7): a positive feedback loop that further increases T3 levels and effects. This is evident in primary hypothyroidism where the high TSH level produces abnormally high serum T3/T4 ratios, 2x higher than in central hypothyroidism (176).

This positive feedback loop is interrupted by T4 monotherapy since it over-suppresses the TSH and thereby over-reduces T4-to-T3 conversion in the thyroid gland and throughout the body. In addition, the higher FT4 levels reduce T3 production by a direct effect upon the deiodinases. Higher FT4 levels suppress D2 activity in tissues like the skeletal muscles (177) which are a major source of circulating T3 (178). The lower TSH and FT3 levels on T4 monotherapy reduce D1 activity, thereby reducing T4-to-T3 conversion and the degradation of rT3. Higher T4 and/or T3 levels induce D3 production (179). D3 reduces T3 levels and effects by catalyzing the conversion of T4 to rT3 and the degradation of intracellular T3 to inactive T2. On T4 therapy serum rT3 levels are higher than in controls, often above the population range (180). They are 50% higher than on DTE titrated to the same normal TSH level (181).

In summary, once-daily T4 monotherapy is an unnatural intervention into a highly complex, tightly regulated system (172). For the reasons discussed, when T4 is dosed to normalize an elevated TSH, it fails to restore serum T3 levels or T3 effects in the tissues of most persons (**Table 10**).

**Table 10:** Why TSHT4Rx does not produce euthyroidism.

Suboptimal TSH secretion/action in many younger and most older persons
Oversuppression of TSH by peak T4 levels with once-daily dosing
Greater sensitivity of HP system to serum T4 than other tissues
Lower TSH reduces thyroidal and peripheral T3 production
Higher FT4 increases D3 activity, converting T4 to rT3 and T3 to T2
Higher FT4 and rT3 reduce D2-mediated peripheral T4-to-T3 conversion
Lower FT3 reduces D1-mediated T4-to-T3 conversion and degradation of rT3
Lack of the thyroid gland’s deiodinases reduces T4-to-T3 conversion
Common deiodinase variants reduce T4-to-T3 conversion

TSHT4Rx does, of course, rescue a patient from severe primary hypothyroidism and may produce optimal results for some patients. It can even be beneficial for some symptomatic “subclinical hypothyroidism” patients who have vigorous T4-to-T3 conversion—when the T4 dose produces significantly higher 24-hour trough FT3 levels (182). Symptom relief with higher T4 doses is strongly correlated with greater global deiodinase activity as evidenced by higher FT3 levels (183). However, T4 doses that produce low-normal, high-normal, or mildly elevated TSH levels usually do not restore symptom scores to those of healthy persons, and can leave FT3 levels low, even when the TSH is low-normal (184).

TSHT4Rx for primary hypothyroidism therefore produces higher FT4 levels and lower FT3 levels than seen in healthy controls (185–193). As a result of this T3 deficit, TSHT4Rx does not eliminate most hypothyroid symptoms and metabolic abnormalities in most patients at any TSH or FT4 levels within or near their reference ranges. TSHT4Rx can leave hypothyroid symptoms as severe and 24-hour urine T3 levels as low as in untreated hypothyroid patients (62). Most patients on TSHT4Rx continue to suffer significant impairment of their psychological wellbeing, health status, and cognitive function compared to healthy persons (194–197). The higher the TSH level, even within its reference range, the worse the symptoms (198).

Patients on TSHT4Rx have a lower quality of life and a higher rate of comorbidities that further degrade their quality of life (199). They have more depression and anxiety, especially when TSH levels are higher in range (200). They are more likely to be taking antidepressants (201). Compared to carefully matched controls, they have higher body mass indices (BMIs) despite reporting lower calorie intake corrected for body weight. They report lower physical activity levels and are more likely to be taking statins, beta-blockers, and antidepressants (202). They have a lower resting energy expenditure (190) and a 21% greater fat mass (189). They have a larger waist circumference, higher BMI, higher total and LDL cholesterol levels, and lower HDL levels (203). They have lower sex hormone-binding globulin levels (193)—a sign of less T3 effect in the liver. They have higher cholesterol levels (also less T3 effect in the liver) unless they are given T4 doses that suppress the TSH (204). They have persistent endothelial dysfunction (205) and higher cardiovascular morbidity (206). Higher T4 doses that lower the TSH to under 2.0mIU/l produce lower cholesterol, homocysteine, and C-reactive protein levels (207), but produce little improvement in hypothyroid symptoms (208). Therefore, the oft-repeated statement that “almost all” patients receiving TSHT4Rx are “well-replaced” is contradicted by almost all of the evidence. It is a myth; a product of the TSH-T4 Paradigm.

### 4.4 Athyreotic patients fare worse

TSHT4Rx produces worse results in patients without thyroid tissue (65). They have lower FT3 levels (209) due to the loss of the gland’s abundant deiodinases and therefore of intraglandular T3 production (172). Post-thyroidectomy, TSHT4Rx leaves them with much lower FT3 levels than controls (210). To restore their serum T3 to preoperative levels requires FT4 levels that are 40% higher than before surgery (39). Restoring both FT3 levels and metabolic markers to preoperative levels requires T4 doses that suppress the TSH to 0.3 to 0.03mIU/l (211). Alleviating hypothyroid symptoms requires T4 doses that produce a low TSH and an above-mid-range FT3 (212).

Patients who were euthyroid prior to thyroidectomy gain 2.2kgs more than controls in the first year (213). Patients who were hyperthyroid gain 10kgs in the first 18 months on TSHT4Rx, whereas those given TSH-suppressive T4 therapy do not gain weight (214). The failure of TSHT4Rx is obvious when a patient’s health and quality of life deteriorate dramatically after thyroidectomy and are restored with T4/T3 therapy (215). I have seen athyreotic patients who were nearly incapacitated by TSHT4Rx. I was able to restore their health and quality of life with DTE titrated by clinical criteria, without regard for the low or suppressed TSH.

### 4.5 Inadequate treatment of central hypothyroidism

The TSH-T4 Paradigm produces even worse results for patients with central hypothyroidism. The TSH should be ignored, but because physicians reflexively associate a low TSH with thyrotoxicity, they often prescribe only enough T4 to produce a low-in-range TSH (216, personal experience). This necessarily leaves the FT4 much lower than in T4-treated primary hypothyroidism (217). Most physicians will just normalize the FT4, leaving the FT3 low in nearly 50% of patients (120). The ATA/AACE recognize this pitfall and recommend keeping the FT4 in the upper half of its reference range (tweaking the paradigm). However, because the TSH is suppressed, thyroidal and peripheral T4-to-T3 conversion are reduced. At the same FT4 level, central hypothyroid patients will produce less T3. Some experts have recommended a T4 dose that raises the FT4 to the top of its reference range and the FT3 to the upper half of its range (216). Others have recommended ignoring the paradigm altogether and attending to the relative FT4 and FT3 levels and indicators of peripheral T3 effect—i.e., signs and symptoms (218).

In T4-treated central hypothyroidism weight gain is common (“hypothalamic obesity”), as are listlessness and other hypothyroid symptoms. The cause is inadequate treatment. The addition of 30 to 60mcgs of T3 to such patients’ “appropriate” T4 doses produced weight loss and alleviated hypothyroid symptoms (219). Increasing endocrinologists’ “empirical” 1mcg/kg T4 doses by 60% improved patients’ metabolic indicators and symptoms. Increasing their T4 doses by 44% and adding a body-weight-adjusted 8 to 12mcgs of T3 produced greater improvements (216).

## 5 Clinical diagnosis of hypothyroidism

### 5.1 Clinical medicine

The alternative to the TSH-T4 Paradigm is clinical thyroidology. Clinical medicine is “the study and practice of medicine in relation to the actual patient; the art of medicine as distinguished from laboratory science” (220). To practice clinical medicine is to acknowledge that the human body is more complex than we understand and that individuals vary greatly in their response to any given serum level, medical condition, or therapy. Our medical knowledge remains rudimentary, our tests limited, and our ideas frequently inadequate or false. There are hundreds of symptoms, disorders and diseases whose causes we do not know and for which we have no cure. We must admit our ignorance and treat patients—not reference ranges, not population correlations, not randomized controlled studies, and not guidelines. Population studies and trials can only inform, they can never replace clinical medicine. To practice clinical medicine is to practice mindfully within the context of all that is known about human physiology, its disorders and their treatment. It requires attention to the patient’s history, symptoms, physical signs, and test results. It requires gathering additional evidence as required, interpreting that evidence, and creating and testing theories of the cause. It may require trials of diagnostic and/or therapeutic interventions. It requires clinical judgment (221).

### 5.2 Best indicators of T3 effect: signs and symptoms

Signs are objective indicators—they can be observed and/or measured by the physician. Signs can be quantitative or qualitative. Some quantitative signs of T3 effect are body temperature, pulse, basal metabolic rate, and tendon reflex relaxation time. Others are serum tests including sex hormone binding globulin, total and LDL cholesterol, lipoprotein(a), and apolipoprotein B (222). Qualitative signs of suboptimal T3 effect include facial and/or ankle myxedema, dry skin, coarse scalp hair, loss of body hair, slow movement, and cold extremities.

Physicians must also attend to the patient’s symptoms. Symptoms are much more sensitive indicators of tissue T3 effect than signs. They are usually present long before signs appear, and often exist in the absence of signs. It is true that, considered individually, many hypothyroid symptoms are non-specific—they can have other causes. However, the greater the number of symptoms that are more specific for hypometabolism/hypothyroidism (e.g., cold intolerance, dry skin, hoarseness, constipation, weight gain, and muscle cramps), the greater the likelihood that hypothyroidism is the cause. The likelihood of hypothyroidism is further increased if the FT4 and/or FT3 levels are low in their ranges or low. It is also increased when the physician cannot find any other explanation for the symptoms. Non-anemic iron deficiency can mimic hypothyroidism and should not be missed (223). It reduces FT4 and FT3 levels and iron supplementation increases them (224, 225). A serum ferritin of <100ng/ml suggests iron deficiency and a ferritin of less than 60ng/ml is a common cause of inadequate improvement with TRT (226).

Because the TSH-T4 Paradigm has caused physicians to dismiss the signs and symptoms of hypothyroidism for decades, their awareness of them has declined. To diagnose hypothyroidism clinically, physicians must familiarize themselves with its protean manifestations. They must not only attend to the patient’s complaints but must actively search for signs and symptoms that could be caused by hypothyroidism. Patients are less aware of symptoms that have come on gradually over time (153) because they have accepted them as their “new normal”. Physicians must therefore tease out chronic symptoms through direct and indirect inquiries, conducted in a neutral manner to avoid leading the patient. Often a patient will present with one complaint, but careful questioning will reveal other hypothyroid symptoms. Many symptoms can be discovered only by inquiring about coping mechanisms (e.g., extra heaters, blankets, laxatives, skin lotions, etc.). Sometimes the physician will learn of a symptom only when the patient reports that it improved or disappeared with treatment.

### 5.3 Best serum indicator of T3 effect: FT4 and FT3 considered together

There is a need for a more reliable serum indicator of T3 status for untreated persons, both for population studies and clinical practice. The TSH is manifestly unfit for this purpose. An FT4 or FT3 level alone provides only part of the picture. The FT4 indicates the availability of the more abundant prohormone, while the FT3 indicates both the amount of T4-to-T3 conversion that is occurring in the tissues and the availability of the active hormone to those tissues that rely upon serum T3 (227). Both must be considered to obtain the most accurate serum estimate of intracellular T3 levels.

To judge whether a patient’s symptoms could be caused by hypothyroidism requires clinical judgment informed by the FT4 and FT3 levels, the exclusion of other causes, and ultimately, a trial thyroid optimization therapy that includes both T4 and T3. (See below.) Persisting improvement on clinically optimized T4/T3 therapy strongly supports the diagnosis.

## 6 Clinical treatment of hypothyroidism

### 6.1 Clinical reference ranges for T4 therapy in primary hypothyroidism

The treatment of hypothyroidism must proceed by clinical criteria first and the FT4 and FT3 levels second. TSH secretion evolved to control the thyroid gland and peripheral deiodinase activity; not to tell doctors what dose of T4 or T4/T3 a person should swallow every morning. Even the most carefully produced untreated TSH, FT4, and FT3 population reference ranges do not apply to treated patients. The timing of the blood draw relative to the T4 or T4/T3 dose strongly affects the results.

In 1986, four experienced clinicians adjusted the once-daily T4 doses of 148 long-term patients based upon clinical criteria alone—signs and symptoms—using a modified Wayne clinical index (36). For those patients judged to be clinically euthyroid, the 2SD reference ranges were as shown in **Table 11**. The TSH level was the least reliable indicator of clinical euthyroidism. The FT4 treatment range was 50% higher than the population range. In contrast, the FT3 treatment range was nearly identical to the conventional range. Therefore, if T4 monotherapy is to be guided by any test it should be the FT3. The breadth of the clinical treatment FT4 and FT3 ranges attests to their high individuality. Laboratories should report similar ranges for T4-treated persons.

**Table 11:** Laboratory vs. clinical T4Rx ranges.

Laboratory Ranges	Clinical T4Rx Ranges
TSH: 0.35–5 mIU/l	<0.1-13.7 mIU/l
FT4: 9–25 pmol/l	12–36 pmol/l (0.93-2.8 ng/dl)
FT3: 2.9-8.9 pmol/l	3.0-8.6 pmol/l (1.95-5.6 pg/ml)

### 6.2 Low or undetectable TSH on TRT does not indicate thyrotoxicosis

The primary impediment to the effective clinical diagnosis and treatment of hypothyroidism is the ATA/AACE’s illogical reliance upon the TSH test and the resulting fear of a low TSH. Almost all physicians believe that a low TSH on replacement therapy has the same implications as does a low TSH in untreated persons in population studies—that it indicates thyrotoxicosis and portends bone loss, cardiac arrhythmias, heart attack, and stroke. Thus, they will not diagnose dysfunctional central hypothyroidism or peripheral resistance when the TSH is normal because effective treatment will suppress the TSH. They also will not try to alleviate a treated patient’s persisting hypothyroid symptoms by raising the T4 dose or adding T3. If the TSH is low on TRT, they reflexively reduce the dose, giving no thought to the nature of the hypothyroidism, the actual FT4 and FT3 levels, or the patient’s signs and symptoms.

TSH deficiency, of itself, has no known pathological effects. It simply reduces intrathyroidal T4/T3 and peripheral T3 production. The fear of a low TSH on TRT arises from population correlations seen in untreated persons, in whom a low TSH is correlated with thyrotoxicosis and its sequelae. However, population correlations have no relevance to any individual, unless proven so, and untreated TSH-population correlations have no relevance to T4- or T4/T3-treated populations. As mentioned, the TSH is oversuppressed by the serum peaks with once-daily oral therapy. Many studies, including many cited in this paper, have found that many patients require T4 doses that produce a low or suppressed TSH to restore T3 levels and effects—to restore clinical euthyroidism.

A treated TSH is not the same as an untreated TSH. Patients with true endogenous “subclinical hyperthyroidism” (i.e., Graves’ disease, toxic multinodular goiter, etc.) can have signs and symptoms of thyroid excess even though their TSH is only slightly low (avg. 0.15mIU/l). Their FT4 and FT3 levels are typically both in the upper thirds of their ranges (228). In endogenous “overt hyperthyroidism,” the TSH is often only partially suppressed when the FT4 and FT3 levels are both high 24hrs/day. The FT3 is usually disproportionately high relative to the FT4. Neither these clinical findings, nor high FT4 and FT3 levels, nor high FT3/FT4 ratios are seen with clinically adjusted T4 therapy that happens to produce a low or undetectable TSH. In thyroidectomized patients, extensive testing found no evidence of thyrotoxicity with T4 and T4/T3 doses that produced an average TSH of 0.1 to 0.3mIU/l (229).

What is true is that T4 or T4/T3 dosing that is excessive for the patient will usually produce a low or suppressed TSH. So, among persons with low or undetectable TSH levels on TRT, there will be more persons who are actually overtreated, an expected population correlation. In a retrospective cohort study of T4-treated patients, very low (<0.1mIU/l) or undetectable TSH levels were associated with a small increase in mortality (230). However, a low or undetectable TSH does not imply overtreatment in any given individual. On the other hand, some patients have medical conditions that make them intolerant of any increase in T3 effect, even when they have relatively low FT4 and FT3 levels and hypothyroid symptoms. (See below.) Some cannot tolerate the normalization of their TSH with T4 or T4/T3 therapy.

The benignity of a low TSH on once-daily T4 therapy for primary hypothyroidism has been acknowledged by some authorities. The UK’s Royal College of Physicians states that a correct dose of T4 may produce a “normal or below normal serum thyroid stimulating hormone concentration” (231). Others have stated, “Some patients achieve a sense of wellbeing only if free T4 is slightly elevated and TSH low or undetectable … it is not unreasonable to allow these patients to take a higher dose if T3 is unequivocally normal” (232). There is also a great deal of data from patients receiving TSH-suppressive T4 therapy (TSHSupT4Rx) for thyroid cancer. They are not usually thyrotoxic, either by symptoms or objective measures. Calorimetry shows no increase in metabolism compared to their pre-surgical state (233). Many still have worse SF-35 mental scores than healthy controls and some have low FT3 levels (234).

I am not arguing that a low or undetectable TSH is a goal of thyroid optimization therapy. It is best if the TSH does not need to be suppressed as it retains thyroid gland activity and improves peripheral T4-to-T3 conversion. It may be possible to avoid TSH suppression in many persons who have vigorous TSH secretion and no peripheral thyroid resistance, especially with multiple daily T4/T3 doses or slow-release T4/T3 tablets that produced more stable levels 24hrs/day. What I am arguing is that the TSH cannot be used to determine the treatment of hypothyroidism. In my experience, many persons on DTE or T4/T3 therapy require doses that completely suppress their TSH level, yet they have moderate FT4/FT3 levels and no signs or symptoms of thyrotoxicosis.

### 6.3 Common concerns about TSH-suppression with TRT

Physicians believe that a low TSH heralds bone loss that may lead to a fracture. TSHSupT4Rx has been associated with a faster loss of bone density in some studies, but not all (235). What matters is the cause. Greater T3 effect increases the metabolic activity of every tissue, including bone. Bone is constantly being remodeled. More T3 effect increases the rate of bone turnover (236, 237). If a person is in a bone-catabolic state, actively losing bone mass, then more T3 effect increases the rate of loss. After age 30 most persons are in a bone-catabolic state largely due to the age-related loss of bone-anabolic hormones; women more so than men (238). When women over age 30 develop primary hypothyroidism, just normalizing their TSH with T4 therapy increases their rate of bone loss for the first 6 months (239). Menopausal women are in a bone-catabolic state due to estradiol deficiency, so they are most likely to lose bone density faster on TSHSupT4Rx (240). Estrogen replacement therapy prevents this excess bone loss (241). The contrary is also true: if a person is in a bone-anabolic state, more T3 effect speeds up the increase in bone density. Adolescent females on TSHSupT4Rx gain bone mass faster than controls (242). A young hypopituitary woman experienced a 20% increase in bone density over 2 years when 50mcgs of T3 was added to her existing T4 and estradiol regimen (219).

Physicians also fear TSH suppression because hyperthyroidism is associated with liver pathology. An early study claimed that T4 doses that produced low TSH or high FT4 levels caused “hepatic damage” indicated by high glutathione-S-transferase levels (26). However, with T4 therapy glutathione-S-transferase and some transaminase levels are far less elevated than in endogenous hyperthyroidism (27). Again, what matters is the cause. Due to the first-pass effect, oral TRT exposes the liver to higher T4/T3 levels than other tissues, causing changes in liver function tests that resemble hyperthyroidism. The liver contains abundant D1 and converts portal T4 to T3 (243). These liver effects are apparently benign. There were no reports of liver pathology in the many decades when patients received higher, clinically optimized T4 and T4/T3 doses.

Physicians also fear TSH suppression because endogenous hyperthyroidism is associated with an increased risk of myocardial infarction, stroke, and thromboembolic disease (244). However, these patients have high FT4 and FT3 levels 24hrs/day; with proportionately higher FT3 levels. They have signs and symptoms of thyrotoxicosis and may have other abnormalities related to the cause of their hyperthyroidism. Such levels, signs, and symptoms are not present in patients on clinically adjusted T4 or T4/T3 doses that happen to suppress the TSH, for the reasons given above. Again, in the pre-TSH era, there was no data suggesting that treated patients had an increased risk of cardiovascular events. A recent review found that T4-treated primary hypothyroidism patients with low TSH levels (0.04-0.4mIU/l) had no increase in cardiovascular events or mortality compared to those with normal TSH levels. Only those with high TSH levels or the lowest TSH levels (<0.04mIU/l) had a statistical increase in morbidity (245). This is, again, an expected population correlation. Overtreatment can be avoided by careful attention to symptoms, signs, and FT4/FT3 levels.

More relevant to thyroid optimization therapy (TOT) is the fact that moderately excessive T3 effect in the heart, as in mild endogenous hyperthyroidism, produces increased heart rate, excessive contractility, impaired diastolic relaxation, and thickening of the walls. These changes are seen in endogenous “subclinical hyperthyroidism” with an average TSH of 0.15mIU/l and higher-in-range FT4 and FT3 levels than controls. Such patients usually have thyrotoxic symptoms (228). These changes can be seen in patients on TSHSupT4Rx when the T4 dose is excessive (246) and there are thyrotoxic signs and symptoms (247). Lowering the T4 dose eliminates the thyrotoxic signs/symptoms and cardiac abnormalities, even though the TSH remains low (avg: 0.1mIU/l) (248). Athyreotic patients on TSHSupT4Rx can have no cardiac symptoms and normal cardiovascular studies even though their average TSH is just 0.03mIU/l and their FT4 is 50% higher than controls. Their FT3 is similar to controls (249). On the other hand, persons with significant atherosclerotic heart disease may not tolerate any increase in T3 levels/effects due to increased cardiac work and resultant worsening of angina or triggering of arrhythmias.

### 6.4 Risk of atrial fibrillation with thyroid optimization therapy

The best reason to fear a low TSH with treatment is also the best reason to fear all TRT, particularly TOT: the risk of atrial fibrillation (AF). This too must be understood in context. Most persons who develop AF are not on TRT or TOT. AF is common; the lifetime risk of AF is 1 in 5 for persons with no risk factors and rises to over 1 in 3 for persons with one or more risk factors (250). The incidence of AF rises with age, affecting 1 in 25 persons over 60 and 1 in 10 persons over 80 (251). Risk factors for AF are common in the population.

AF risk is clearly related to T3 status. It has been reported with hypothyroidism (252) but is much more frequently seen with higher FT4 and FT3 levels and hyperthyroidism. More T3 effect increases automaticity and trigger activity in the pulmonary vein myocytes that initiate AF (253). In untreated persons, the risk of AF rises with higher FT4 levels within the population range (254, 255) and with lower-in-range or low TSH levels (256). Therefore, any increase T3 effect with T4 or T4/T3 therapy entails a risk of triggering AF in susceptible patients. It does not require overtreatment but is more likely with overtreatment. AF is more likely to occur with a suppressed TSH—an expected population correlation.

Clinically optimized T4 or T4/T3 therapy thus necessarily entails a higher risk of AF than less effective TSH- or FT4-normalizing therapy. The TSH-T4 Paradigm minimizes the risk of triggering AF in susceptible persons, but at the costs of widespread underdiagnosis and undertreatment. This is neither scientifically nor ethically justifiable. Mild-to-moderate hypothyroidism has its own medical risks, and patients want to feel and function as well as possible. The physician can resolve the ethical and legal dilemmas surrounding AF by obtaining informed consent for TOT. The physician and patient should together weigh the patient’s risk of AF against the potential health and quality-of-life benefits of optimal T3 effect. The patient should be given the right to choose. Fortunately, for most persons under age 60, the risk of AF is low, even with overtreatment. In persons over 60 and/or with risk factors, the physician can minimize the risk of AF by going “low and slow” with T4 or T4/T3 dosing. Fortunately, AF that is induced by TRT usually resolves with reducing the dose, except in older patients with significant underlying heart disease (257).

## 7 Hypocortisolism and inflammation

*It is important to emphasize that none of the tests* [for hypocortisolism] *will be perfect and therefore, clinical judgment should prevail in patients with significant symptoms and apparently normal or equivocal biochemical data* (258).

It is very convenient for physicians to limit thyroidology to one test, the TSH, and ignore the patients’ symptoms and all other aspects of their endocrine system, physiology, and medical conditions. However, that is not possible. The thyroidal system is part of an integrated neuro-endocrine-immune system. T3 has especially powerful interactions with cortisol. These interactions affect how hypothyroidism and hyperthyroidism present and can cause anomalous positive and negative reactions to T4, T4/T3, and T3 treatment. Knowledge of these interactions is necessary to treat patients and interpret studies, including T4/T3 combination therapy studies.

### 7.1 T4/T3 therapy can unmask hypocortisolism

One interaction between T3 and cortisol is well known: TRT can unmask or worsen “adrenal insufficiency” (AI). However, relative hypocortisolism, without adrenal gland or HP damage, can arise from many causes and cannot be ruled out by a normal AM serum cortisol (ref. range: 5 to 20mcg/dl (259). It also cannot be ruled out by a normal adrenocorticotropic hormone (ACTH) stimulation test (260–264). Relative hypocortisolism and/or inflammation is suggested when TRT rapidly causes worse fatigue, achiness, and/or brain fog, or worsening of an existing inflammatory disease. As with hypothyroidism, diagnosing relative hypocortisolism requires attention to the patient’s history, signs and symptoms, relative serum and saliva cortisol levels, responses to TRT, and ultimately, response to a trial of hydrocortisone.

### 7.2 Cortisol-T3 interactions

Cortisol and T3 are the most powerful hormones in the endocrine system, and they counteract and support each other’s levels and effects in various ways (**Table 12**). Their interactions are so powerful that the balance between them is as important as the absolute status of either. Together their actions and interactions play a large role in our mental and physical stamina and well-being. Both hormones affect the secretion and/or effectiveness of other major hormones (e.g., the gonadal steroids and growth hormone). Both have large roles in glucose production, availability, and utilization. Mitochondria have both T3 and cortisol receptors (265, 266); both hormones are required to ensure optimal mitochondrial energy production (267). A relative deficiency of one or both produces a hypometabolic state accompanied by fatigue, depression, muscle/joint pain, and cognitive dysfunction.

**Table 12:** T3-Cortisol interactions.

Cortisol is required to produce T3 effect and to tolerate T3 effect.
T3 stimulates ACTH and cortisol production.
T3 increases cortisol utilization and metabolism.
Oral T3 has greater effects on cortisol production and metabolism.
More T3 effect can worsen or improve hypocortisolism.
Hypothyroidism can cause central hypocortisolism.
Cortisol reduces TSH secretion and T4-to-T3 conversion.

Most of T3’s effects are genomic and occur on a time scale of weeks and even months, but higher T3 levels/effects immediately stimulate more ACTH and cortisol production (268, 269) while also increasing cortisol utilization and metabolism (270). Oral T3 has greater effects than endogenous T3 on ACTH-cortisol production due to its high peak levels and on cortisol utilization and metabolism due to its strong first-pass effects in the liver. Almost all positive and negative clinical responses that occur within hours or days from the initiation of T4 and/or T3 supplementation are due to alterations in cortisol levels and effects or T3’s rapid immune system stimulating effects. Persons with relative hypocortisolism may improve rapidly when oral T3 succeeds in raising cortisol levels and effects. On the contrary, if T4 or T4/T3 increases cortisol metabolism more than its production, they may be unable to tolerate necessary thyroid supplementation.

In the absence of cortisol, the affinity of thyroid receptors for T3 is reduced by 50% (271). In marked hypocortisolism, endogenous T4 and T3 fail to suppress TSH production, resulting in an elevated TSH (“subclinical hypothyroidism”). On the other hand, higher cortisol levels and effects reduce T3 levels and effects by reducing TSH secretion (272) and T4-to-T3 conversion (273). In hypothyroidism ACTH and cortisol production are reduced (274), sometimes so much as to produce symptomatic central hypocortisolism (275).

### 7.3 Hypocortisolism and the female sex

Most persons who suffer from a relative hypocortisolism are female. Women have lower cortisol levels and effects, and lower cortisol responses to stress than men (276–283). This is a sufficient explanation for their greater resistance to infections and their much higher incidences of disorders and diseases that are associated with hypocortisolism (allergies, autoimmune diseases, chemical sensitivities, anxiety, achiness, etc.).

Women’s relative hypocortisolism is a sufficient explanation for their much higher incidences of Hashimoto’s and Graves’ diseases (15:1 and 7:1 respectively). Autoimmune thyroid disease (ATD) has been correlated with hypocortisolism (259, 284–286) and other autoimmune diseases (287). Those with Hashimoto’s thyroiditis suffer from constitutional symptoms caused by the autoimmune process and its accompanying inflammation, as shown by the improvements that they experience with thyroidectomy (288, 289). Relative hypocortisolism is therefore common among women with ATD—who comprise most of the subjects in studies of TRT, including the T4/T3 studies. Women’s relative hypocortisolism is a sufficient explanation for the fact that women are the majority of persons who suffer from unexplained fatigue, pain, cognitive dysfunction, anxiety, gastrointestinal dysfunction, and other constitutional symptoms, have mild TSH elevations, are diagnosed with hypothyroidism, have anomalous responses to TRT, and are dissatisfied with TRT.

## 8 Safety and efficacy of T4/T3

Producing optimal clinical results for patients with TRT is a very different exercise than just normalizing serum TSH, FT4, or FT3 levels. Without a trial of TOT, physicians cannot rule out hypothyroidism as a contributor to the patient’s ongoing symptoms. If TOT does not help or makes the patient feel worse, then physicians know to search for another cause: hypocortisolism, inflammation, autoimmune disease, chronic infection, etc. For patients with persisting hypothyroid symptoms on TSHT4Rx, physicians can increase the T4 dose, without regard for a low or suppressed TSH. However, studies show that this will usually fail to restore euthyroidism in all tissues and may cause hyperthyroidism in tissues with high D2 activity like the heart (290).

### 8.1 The need for T3

The thyroid gland secretes T3 and T4, so to replicate normal physiology physicians must include T3 (291). T4/T3 therapy is logically and scientifically necessary to restore optimal T3 effects in all tissues. Providing T3 with T4 keeps FT4 levels lower and thus avoids T4-mediated downregulation of D2 and upregulation of D3. Providing T3 induces greater D1 activity and therefore T4-to-T3 conversion. It compensates for the over-reduction or suppression of TSH secretion due to peak levels and resultant over-reduction in TSH-induced T4-to-T3 conversion. It compensates for the lack of thyroidal deiodinases in athyreotic patients and the lack of TSH in central hypothyroidism. Providing T3 keeps the T4/T3 ratio lower and so improves sensitivity to both T4 and T3, i.e., prevents and treats genetic and acquired forms of thyroid resistance including common deiodinase variants. T3 also helps to stimulate ACTH/cortisol secretion and so can prevent or treat relative hypocortisolism. Unlike low doses of T4 that can reduce T3 effect, providing T3 in a 4:1 T4/T3 ratio guarantees that even low doses increase T3 effect long term, providing reliable information to the physician and patient. T4/T3 therapy has other practical advantages. It improves compliance because patients experience therapeutic effects more quickly and quickly feel a loss of effect if they omit doses (18). In summary, it makes sense to give all patients T4/T3 combination therapy from the start. There will be some patients, however, who will not tolerate oral T3 and/or for whom T4 will work best.

The ATA/AACE guidelines’ authors suggest that oral T3 supplementation is unsafe due to the supraphysiological peak T3 levels after each dose. They provide no evidence. In fact, the T3 peaks have never been shown to produce any health problems. In my experience, the T3 peaks with once daily 4:1 T4/T3 therapy cause problems only for a few persons with hypocortisolism, inflammation, or cardiac disease. The guidelines’ authors also express concern that T3 levels vary throughout the day and “cannot be easily monitored”. In fact, they do not need to be monitored. Oral T3’s pharmacokinetics are well known. T3 levels peak 2.5hrs after an oral dose and have a half-life of around 22 hours (168). With a substantial T3 dose, FT3 levels are usually high at their peak and fall gradually to midrange or low in range at the 24-hr trough. The ATA/AACE authors also assert that we cannot give patients T3 because we do not have slow-release T3 tablets or capsules that can perfectly replicate the more stable 24-hr TSH, FT4, and FT3 levels of a normal person. It may be best to replicate such levels, however, the fact that we cannot do so does not imply that we cannot prescribe any T3.

T3 is our thyroid hormone. T3 supplementation can cause problems only if its dose is excessive (16). T3 is feared precisely because it is efficacious—providing T3 with T4 assures greater thyroid effect, and therefore a greater risk of overdosing and/or of causing problems in susceptible persons (e.g., bone loss, AF). Overdosing is what should be avoided, not T3. A 17-year observational study found that, compared to T4 therapy, patients receiving T4/T3 and T3-only therapies had no increased risk of cardiovascular disease, atrial fibrillation, or fractures (292). A more recent retrospective study found that DTE and T4/T3 treatment, adjusted to a normal TSH, produced no adverse effects or events (293).

### 8.2 Human thyroidal T4/T3 ratio

What is the ideal T4/T3 ratio for TRT for most persons? It is commonly stated that the human thyroidal T4/T3 output ratio is a high 17:1. This belief is based upon a single study in 1990 that involved injections of radio-labeled T4 and T3 in euthyroid persons who were given Lugol’s solution twice daily (294). It is known that such an acute excess of iodine substrate causes the thyroid gland to preferentially synthesize T4 over T3. It inhibits thyroidal D1 activity and D1 mRNA expression, reducing T3 output (295). The human ratio is certainly much lower than this. A 9:1 T4/T3 ratio given to thyroidectomized patients produced a high PBI—i.e., contained an excessive amount of T4. A 3.3:1 ratio produced better clinical results and a normal PBI (18). DTE’s 4:1 ratio normalizes rT3 levels but produces lower FT4 levels than controls with once-daily dosing. The human thyroid T4/T3 output ratio is probably between 6:1 and 8:1. The ratio can be determined as it was in rats, by giving thyroidectomized persons continuous infusions of various ratios of T4 and T3. However, the ideal ratio for once-daily oral dosing will likely be lower than the thyroidal output ratio due to the over-reduction of the TSH by peak levels. It will also vary among patients.

### 8.3 Existing studies support T4/T3 therapy

In the past 54 years, many studies have compared T4 monotherapy with T4/T3 combination therapy. These studies provide abundant detailed information about the serum levels, symptom scores and physiological parameters of persons on T4 monotherapy and various combinations of T4 and T3. Most studies involved arbitrary substitutions of some amount of T3 for some amount of the patient’s usual T4 dose, without regard for the patient’s symptoms or serum FT4 and FT3 levels. Arbitrary substitutions are problematic. They will result in overtreatment or undertreatment for some persons. While oral T3 (as monotherapy) is generally about 3.5 times more potent than T4 by weight in its ability to lower the TSH (296), their relative oral potency can differ by a factor of 3 in different persons and with different doses (297); i.e., from ~1:1 to 10:1. Using the 3.5:1 ratio, the arbitrary substitutions ranged from 1-for-1mcg oversubstitution to 1-for-5mcg undersubstitution—the latter being most common and often produced an effective reduction in dose as evidenced by a higher TSH. In some studies, T4/T3 was allowed to produce different TSH levels; in a few the T4 doses were adjusted to maintain a similar TSH.

Randomized trials with arbitrary substitutions of T3 for T4 cannot override our knowledge of thyroid physiology or the need for individualized treatment based upon clinical judgment (183). Consider that if one-half of the patients in a study improve and one-half deteriorate, the net result is no change. To obtain useful information one must separate the two groups and determine why each reacted as they did. Due to individual variations in T4-to-T3 conversion, cortisol status, autoimmune disease, and other factors, the same FT4/FT3 levels that produce benefits in some persons will produce negative symptoms in others. Furthermore, hypocortisolism and/or inflammation are more common in women with ATD—the majority of subjects in most T4/T3 studies. They can be sensitive to changes in their TRT.

Reviews and meta-analyses of the T4/T3 studies have concluded that they do not support replacing T4 monotherapy with any form of T4/T3 combination therapy (298–302). However, the metanalyses failed to account for the flaws and heterogeneity of the studies. To assess the T4/T3 studies’ implications, each must be analyzed individually, and the analysis must not be based upon the TSH-T4 Paradigm. Both significant and non-significant changes must be considered, along with patient preference. I provide such an analysis of each study here, informed by my clinical experience with attempting to optimize T3 status clinically with T4/T3 therapy (mostly DTE) in more than a thousand patients over 20 years. See **Table 13** for a brief description of each study and its results. See **Supplementary 2** for detailed summaries of and commentaries on each study.

**Table 13:** Studies of T4/T3 combination therapy vs. T4 monotherapy.

Study	Study type	Female %	Duration	Result	Patient preference	Notes
Smith 1970 (32)	DB/CO, Pre-TSH, Large 1-for-1 oversub (20mcgs T4 replaced with 20mcgs T3, for each 100mcgs T4).	85	2 mos	T4 superior	T4 by 1.7:1	Large oversub in patients on clinically optimized T4 doses of 200 to 300mcgs, T4/T3 overdosing resulted in adverse effects for many.
Cooke 1982 (303)	Open label, T3 add-on for depressed patients on 125 to 200mcgs T4 with low TSH levels	88	3–6 mos	T4/T3 superior	T4/T3 by 7:2	Addition of 15-50mcgs T3 to strong T4 doses (and low TSHs) provided long-term relief from depression in 6 of 9 patients.
Bunevičius 1999 (304) 2000 (305)	DB/CO, 1-for-4 equipotent sub, similar low-in-range TSH, 50:50 ATD/Thyroid cancer	93, 100	5 wks	T4/T3 superior	T4/T3 by 10:1	Scales improved on T4/T3, Athyreotic patients experienced the greatest improvements in cognition and mood on T4/T3
Baisier 2001 (62)	Open label, Clinically adjusted DTE-for-T4 sub.	90	27 mos	DTE superior	Not stated	TSHT4Rx patients had urine free T3 levels in hypothyroid range, DTE increased urine free T3 levels and improved symptoms by 2.5x.
Bunevičius 2002 (306)	DB/CO, ATD with subtotal thyroidectomy, 1-for-5 undersub, TSH similar	100	5 wks	T4/T3 superior	T4/T3 by 3:1	Even with undersub, T4/T3 at similar low-in-range TSH alleviated symptoms of both hypo- and hyperthyroidism.
Walsh 2003 (307)	DB/CO, ATD, 1-for-5 undersub → ↑TSH (3.1 vs. 1.5), i.e., T4/T3 undertreatment	92	10 wks	T4 superior	T4 by 1.3:1, If TSH similar, T4/T3 by 1.2:1	Effective dose reduction. Slightly worse symptom scores on T4/T3. Some had nausea, anxiety with T4/T3 c/w hypocort./inflam.
Sawka 2003 (308)	DB, Depressed patients on TSHT4Rx, Avg, 1-for-3.4 equisub→similar normal TSH	90	15 wks	T4/T3 superior	Not stated	On T4 TSH declined, On T4/T3 TSH rose, Both groups improved, but T4/T3 produced greater non-sig improvements in scales.
Clyde 2003 (309)	DB, 1-for-3.3 equisub with 15mcgs T3→similar midrange TSH.	77	4 mos	No difference	Not stated	Both groups improved. One patient dropped out with hypocortisol symptoms on T4/T3.
Siegmund 2004 (310)	DB/CO, Small (7.5mcg T3) 1-for-1 oversub→lower or undetect, TSH, 14:1 T4/T3 ratio.	81	12 wks	No difference	Not stated	TSH undetectable in more pts. on T4/T3, Non-ATD pts, improved, but some ATD pts, had adverse effects c/w hypocortisol/inflammation.
Escobar-Morreale 2005 (311)	DB/CO, 1-for-5 undersub and 1-for-1.67 oversub→higher and lower TSH, Controls included	100	8 wks	T4/T3 superior	T4/T3 by 9:1	Only 1-for-1.67 oversub normalized the Zulewski scale. All pts, had lower QOL and neurophysiological test results than controls.
Appelhoff 2005 (312)	DB, ATD, T4/T3 ratios of 10:1 (equisub), and 5:1 (oversub)	85	15 wks	T4/T3 superior	T4/T3 by 3:1	Patients most preferred the 5:1 ratio oversub that produced low TSH levels and weight loss.
Rodriguez 2005 (313)	DB/CO, ATD, 1-for-5 undersub→ ↑TSH (5.6 vs. 2.7 on T4)	83	6 wks	No difference	T4/T3 by 12:7	Many symptoms worsened with undersub yet patients preferred T4/T3 and lost 2.3 lbs.
Saravanan 2005 (314)	DB, 1-for-5 undersub→↑TSH (2.19 vs 0.76 on T4)	84	12 mos	No difference	Not stated	Despite undersub T4/T3 superior by QOL scale at 3mos due persisting T4 levels/effects, but not at 12mos.
Regalbuto 2006 (229)	DB/CO, Post-thyroidectomy for cancer with low TSH on T4, Fixed 3.5:1 T4/T3 ratio, similar low TSH	85	6 mos	No difference	1:1	Extensive physiological and neuropsychiatric. testing found no thyrotoxicity or adverse effects with low TSH and a low 3.5:1 T4/T3 ratio.
Slawik 2007 (216)	DB/CO, Central, hypothyroidism, avg, 1-for-1 oversub vs. 60% higher weight-adjusted T4 dose	72	5 wks	T4/T3 superior	Not stated	T4/T3 group had greater improvements with less adverse effects, but authors feared that high T3 peaks @2hrs indicated thyrotoxicity.
Nygaard 2009 (315)	DB/CO, 1-for-2.5 oversub, T4 adj. to produce same low-normal TSH	93	12 wks.	T4/T3 superior	T4/T3 by 3:1	Higher 20mcg T3 dose, Marked improvements in scores with no more adverse effects with 4:1 T4/T3 ratio—like DTE.
Valizadeh 2009 (316)	DB, 1-for-4 equipotent sub, T4 adj. to same normal TSH	80	16 wks	T4/T3 superior	Not stated	T4/T3 group had non-sig improvements in 3/4 GHQ-28 scores and sig improvement in anxiety/insomnia. On T4 both worsened.
Hoang 2013 (181)	DB, DTE-for-T4 sub, adjusted to same normal TSH	78	16 wks	DTE superior	DTE by 2.6:1	DTE produced weight loss, lower symptom scores, lower rT3, no adverse effects.
Pepper 2014 (51)	Open label, Clinically adj. DTE after failure of clinically adj. T4. Avg. TSH similar in both groups	93	Min. 4 wks	DTE superior	DTE by 3.5:1	TSH was low in 50% of patients in both arms. No increase in adverse effects was seen with DTE in persons over the age of 65.
Kaminski 2016 (317)	DB/CO, Fixed dose T4/T3 5:1 ratio. 50% mix of 1-for-5 undersub and 1-for-3.5 equisub	94	8 wks	No difference	Not stated	Avg, TSH higher in the T4/T3 group. Both groups improved. T4 group received higher effective dose during study. No adverse effects
Tariq 2018 (293)	Open label TSH-normalizing DTE or T4/T3 after failure of TSHT4Rx	95	27 mos	DTE and T4/T3 superior	DTE: 93% T4/T3 89%	Pts, followed by endocrinologists. Marked improvements reported by almost all patients on both DTE and 15:1 T4/T3.
Heald 2021 (52)	Open label DTE-for-T4 sub after failure of TSHT4Rx. Clinical dose adjustment	90	6 mos	DTE superior	DTE: 90%	Marked improvements in QOL scores, greater improvement on >120mgs DTE. Clinical treatment TSH was <0.01 to 8.5mIU/L
Shakir 2021 (53)	DB/CO TSH-normalizing study comparing T4, DTE and a 10:1 T4/T3 combination	77	22 wks	DTE and T4/T3 superior	DTE: 45% T4/T3 32% T4 23%	Only the most symptomatic T4 patients had significant improvements on DTE and T4/T3. They probably had poor T4-to-T3 conversion.

TSH units, mIU/l; sub, substitution; sig, significant; ATD, autoimmune thyroid disease; QOL, quality of life; DB, double blinded; CO, crossover.

Considering both the statistically significant and non-significant trends and patient preference, the 23 T4/T3 combination studies strongly favor combination therapy. This is despite frequent T4/T3 underdosing as evidenced by higher TSH levels, and occasional T4/T3 overdosing. Fifteen of the 23 studies favored T4/T3 therapy when all results are considered, and only two favored T4—a 7.5:1 ratio in favor of T4/T3. One study that favored T4 involved 1-for-1 oversubstitution and obvious overdosing in patients already receiving high, pre-TSH, clinically optimized T4 doses (Smith). The other involved 1-for-5 undersubstitution and obvious underdosing, indicated by a 2x higher average TSH. Even in that study, if the TSH rose by <0.99miU/L, patients preferred T4/T3 by a small margin (Walsh).

In 6 of 23 studies there was no overall difference in the results. Three of the six neutral studies used 1-for-5 undersubstitution (Rodriquez, Saravanan, Kaminski) and one used 1-for-1 oversubstitution (Siegmund). In the 15 studies in which subjects’ preference was assessed, they preferred T4/T3 in 12 (80%). They sometimes preferred T4/T3 even with 1-for-5 undersubstitution and higher TSH levels. For certain, the T4/T3 studies show that including some arbitrary amount of T3 is not a magic bullet; it doesn’t guarantee better results in all cases. However, it usually produces better results, especially with equipotent 3.5:1 substitutions and lower T4/T3 ratios.

Only one T4/T3 study included healthy controls and it found that most of the T4 and T4/T3 treated patients were more hypothyroid than controls by subjective and objective scales. Scales improved to those of controls only with T3 oversubstitution that suppressed the TSH (Escobar-Morreale). No other studies included controls, so this raises the probability that most patients with normal or even low TSH levels in both the T4 and T4/T3 arms of most of the studies were undertreated. In only three studies was the T4/T3 regimen (DTE) adjusted according to clinical criteria without regard for the TSH (Baisier, Pepper, Heald). Two of those studies compared clinically adjusted T4 and DTE therapies (Baisier, Pepper) and both found DTE to be superior.

### 8.4 Conclusions from the T4/T3 studies

Most of the T4/T3 studies’ designs and interpretations were vitiated by errors, many of which are encountered in other studies of TRT performed within the TSH-T4 Paradigm. Some of these are discussed in **Supplementary 2**. From the standpoint of the clinical paradigm and the scientific evidence here presented, I submit that the following conclusions can be drawn:

- T4/T3 treatment is both efficacious and safe, except with overdosing or underdosing. The T4/T3 studies have disproved the idea that including T3 is somehow dangerous.
- Supraphysiological peak T3 levels after T3 doses do not produce any deleterious effects or negative symptoms in most patients.
- T4/T3 treatment usually produces superior results except when the T4/T3 dose is clearly excessive or inadequate; the latter often indicated by a much higher TSH. Even at significantly higher in-range TSH levels, T4/T3 sometimes produces the same clinical benefits as T4 monotherapy (Saravanan), attesting to its physiological benefits.
- Primary hypothyroid patients usually feel best, and/or obtain the best symptom or physiological test scores on T4 and/or T4/T3 regimens that produce a low or suppressed TSH (Cooke, Escobar-Morreale, Appelhof). They can have no signs or symptoms of thyrotoxicosis, even with extensive testing (Regalbuto).
- Athyreotic and central hypothyroid patients are most likely to experience benefits with T4/T3 combination therapy (Bunevičius, Slawik; Regalbuto is the outlier).
- Studies that recruited patients who are dissatisfied with TSHT4Rx tended to show more positive effects with T4/T3 therapy (Baisier, Pepper, Heald, Tariq).
- The best clinical results were obtained with DTE adjusted by clinical criteria (Baisier, Pepper, Heald). Clinically optimized DTE doses frequently result in a suppressed TSH (Pepper, Heald).
- Persons with ATD tend to have higher FT3 levels on treatment, and some do not tolerate greater thyroid effect on T4/T3, even though clearly not overtreated (Clyde, Siegmund).

In sum, the existing studies indicate that T4/T3 therapy is not harmful and is superior to T4 monotherapy for most patients. Because T4/T3 therapy is more physiological and effective, it should be considered the preferred treatment for hypothyroidism. There is no rational justification for more arbitrary substitution or TSH-normalizing T4/T3 studies. The only meaningful study of T4/T3 vs. T4 monotherapy is a comparison of clinically adjusted dosing of each by experienced clinicians and/or according to hypothyroid symptom scales and patient preference, without regard for the TSH level.

## 9 Conclusion

The 2012 ATA/AACE guidelines for the treatment of hypothyroidism were a reiteration of the TSH-T4 Paradigm from the 1970s. This old, arbitrary, ineffective, laboratory-based paradigm must be replaced by a clinical, patient-centered paradigm that incorporates all current knowledge. Hypothyroidism must be properly defined as insufficient T3 effect in some or all tissues. Diagnosis and treatment must be guided by the most sensitive criteria: the patient’s signs and symptoms first and the relative FT4 and FT3 levels second. T4/T3 therapy is superior and should be favored. DTE’s 4:1 ratio worked well for many decades and still appears to work better than higher T4:T3 ratios for most people. If a person has hypothyroid symptoms for which there is no other medical explanation and has relatively low FT4 and/or FT3 levels, then he/she deserves a trial of gradually increasing T4/T3 doses with careful monitoring for both improvements and evidence of overdosing. If the latter occurs, or if the FT4 and FT3 levels are rather high with treatment, the dose should be reduced and the result monitored. The optimal T4/T3 dose is the lowest dose that eliminates hypothyroid signs and symptoms without producing any signs or symptoms of excess T3 effect. The TSH level provides some information but cannot be relied upon for either diagnosis or treatment. A suppressed TSH with treatment is not proof of thyrotoxicosis.

Hypothyroidism can cause a wide variety of symptoms and disorders and so must be ruled out in all cases of unexplained symptoms. Physicians’ ability to understand hypothyroidism and willingness to provide a trial of T4/T3 thyroid optimization therapy will improve the lives of countless patients around the world. It will also open the door to a greater understanding of the underlying causes of fatigue, achiness, cognitive dysfunction and other “functional” disorders. If a trial of T4/T3 thyroid optimization eliminates patients’ symptoms indefinitely, then they were indeed suffering only from a lack of T3 effect in their tissues—for whatever reason. If their symptoms improve only temporarily or are worsened by T4/T3 optimization therapy, then physicians gain additional clues regarding the cause. They must consider hypocortisolism, chronic infection, autoimmune disease, iron deficiency, toxins, genetic disorders, etc. By performing additional tests and/or therapeutic trials for these other conditions, they will be able to help more suffering people. An effective thyroidology therefore will not only optimize the diagnosis and treatment of all forms of hypothyroidism but will open the door to greater understanding of endocrinology and of the causes of many currently unexplained disorders.
