# Correction: Clinical thyroidology: beyond the 1970s’ TSH-T4 Paradigm

**DOI:** 10.3389/fendo.2025.1656676

**Published:** 2025-08-14

**Authors:** Henry H. Lindner

**Affiliations:** Private Practice, Tunkhannock, PA, United States

**Keywords:** clinical medicine, deiodinases, guidelines, hypocortisolism, hypothyroidism, paradigm, reference range, T4/T3 combination therapy

In the published article, there was an error. A correction has been made to the last sentence of section 1 **Introduction**, paragraph 1. This sentence previously stated:

“Optimal T3 effect is essential to every aspect of human development, health, and vitality, including the function of the rest of the immune system.”

The corrected sentence appears below:

“Optimal T3 effect is essential to every aspect of human development, health, and vitality, including the function of the rest of the endocrine system.”

The publisher apologizes for this error and states that this does not change the scientific conclusions of the article in any way. The original article has been updated.

