# Supplementary material for: Clinical thyroidology: beyond the 1970s’ TSH-T4 Paradigm
Source: Front Endocrinol (Lausanne). 2025 Jun 24;16:1529791. doi: 10.3389/fendo.2025.1529791 (PMC12234311; doi:10.3389/fendo.2025.1529791)
Supplement: Supplementary file 1 [file SupplementaryFile1.pdf]

A statistical analysis of the effects of exogenous thyroxine (**T4**) and triiodothyronine (**T3)** dose changes on TSH concentration over a period of 6 years in a 64 year old Asian male with primary hypothyroidism

Stan Altan, Ph.D.

May 28, 2018

Outline

- Patient description and objective
- Data description (design, primary database)
- Statistical modelling strategy (3 models)
- Results from 3 models
- Summary

Patient description and objective

- Patient
  - 64 year old Asian male at initiation of study 2011, normal height and weight
  - Diagnosis: primary hypothyroidism, possibly a consequence of radiologic dye adverse reaction
  - Concomitant conditions: Low testosterone, low ferritin, elevated MCH and MCV
- Objective – use an up-down dose design to achieve a steady state TSH level of 0.50

Data description

- Data collected 12/2011 – 9/2017
- Variables
  - Date
  - No.doses/day
  - AM NDT gr.
  - PM NDT gr.
  - Total T4 dose (mcg)
  - TSH uIU/mL
  - fT3 pg/mL
  - fT4 ng/dL

Date	#Dose /Day	NT-AM Dose (gr)	NT-PM Dose (gr)	T4 dose (mcg)	TSH (uIU/mL)	FT3 (pg/mL)	FT4 (ng/dL)
12/20/2011	1	0.75	0	0	3.85	2.9	0.8
2/24/2012	1	1	0	12.5	0.1	4.1	1.2
4/9/2012	1	0.75	0	0	0.44	3.4	0.9
6/1/2012	1	0.5	0	12.5	5.87	2.6	0.8
9/17/2012	2	0.5	0.25	0	3.65	2.7	0.8
11/3/2012	2	0.75	0.25	0	5.1	2.6	0.93
12/17/2012	2	0.75	0.25	25	1.46	3.1	1.06
1/24/2013	2	1	0.25	0	2.29	2.7	0.74
3/23/2013	2	1	0.25	25	0.112	3.6	1.12
5/28/2013	2	1	0	0	4.27	2.5	0.68
7/15/2013	2	1	0.25	12.5	1.3	2.9	0.87
9/11/2013	2	1	0.5	12.5	0.232	3	0.9
12/5/2013	2	1.25	0.5	0	0.372	2.3	0.73
5/20/2014	3	0.75	0	37.5	4.12	2.4	0.88
7/29/2014	3	0.875	0	50	2.23	2.5	0.93
9/29/2014	3	1	0	25	2.1	2.4	0.93
12/17/2014	1	1	0	50	0.202	3.1	1.11
12/17/2014	1	1	0	50	0.2	3.1	1.11
2/23/2015	1	0.75	0	72	0.146	2.8	1.09
4/14/2015	1	0.5	0	94	0.06	3	1.32
5/28/2015	2	0.25	0.25	94	0.234	2.7	1.15
11/3/2015	2	0.25	0.25	75	1.81	2.8	1.19
1/15/2016	2	0.25	0.25	75	1.13	2.8	1.13
5/5/2016	2	0.25	0	75	1.03	2.7	1.22
9/7/2016	1	0.5	0	75	0.437	2.7	1.19
12/3/2016	1	0.5	0	50	1.03	2.9	1.42
3/6/2017	2	0.25	0.25	50	4	2.4	1
5/10/2017	2	0.25	0.25	37.5	2.04	2.7	1.08
9/26/2017	3	0.5	0	75	3.66	2.4	1.13

Statistical analysis modelling strategy

- 3 Models - TSH or log2(TSH) function of Total T4 dose, T3 dose, Number. of doses/day :
  1. linear, interaction and quadratic terms
  2. linear terms
  3. Log_2 (TSH) linear terms

Table of model description – x indicates inclusion of term							
Model	Response	doses / day	T4 Dose	T3 Dose	T4*T3	T4 ** 2	T3**2
1	TSH	X	X	X	X	X	X
2	TSH	X	X	X			
3	Log_2TSH	X	X	X			

Results of Models 1-3

- Model 1 :
  - Significant linear terms for Total T4 dose, T3 dose, Number of doses/day based on Type 1 SS, no significant curvature term.

- Model 2
  - R^2=0.63
  - MSE = 1.26
  - Normality p>0.15

Model 2 Parameter Estimates				
**Parameter**	Estimate	Std. Error	t Value	Pr > |t|
**Intercept**	5.05	1.02	4.97	<.0001
**T4_Total(mcg)**	-0.03	0.01	-5.55	<.0001
**T3_Total(mcg)**	-0.25	0.07	-3.5	0.0018
**Dose**	1.09	0.32	3.44	0.0021

- Model 3
  - R^2=0.74
  - MSE = 1.16
  - Normality p<0.01

Model 3 Parameter Estimates				
**Parameter**	Estimate	Std. Error	t Value	Pr > |t|
**Intercept**	2.74	0.97	2.81	0.009
**T4_Total(mcg)**	-0.05	0.01	-6.39	<.001
**T3_Total(mcg)**	-0.30	0.07	-4.42	<.001
**Dose**	1.71	0.31	5.61	<.001

Graph of TSH vs Total Suppressive dose* by Number of Doses/day

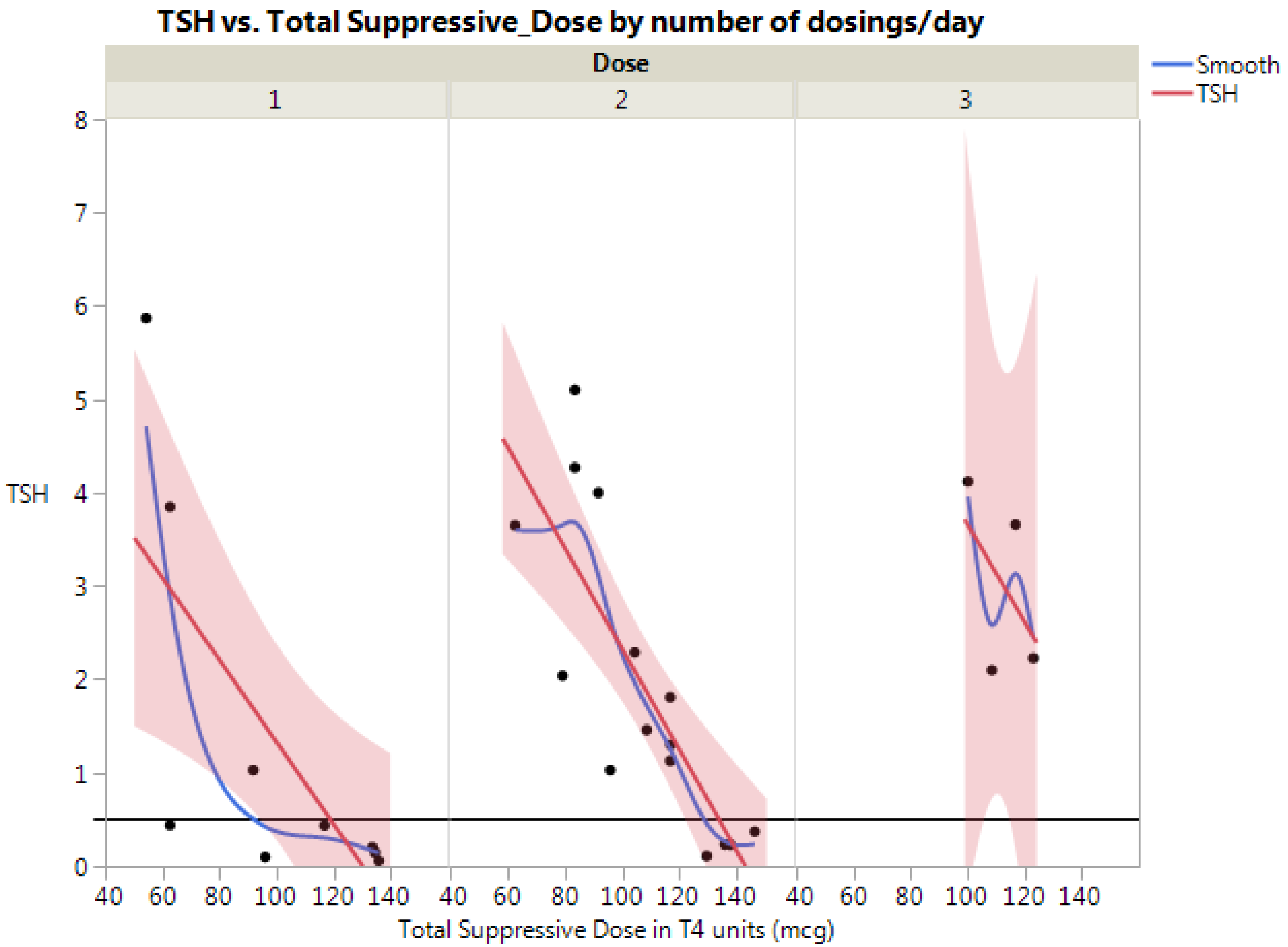

* Total suppressive dose (mcg) = Total T4 (mcg) + 5.1 * Total T3 (mcg)

Summary

- Statistically significant relationship between TSH concentration and Total thyroxine and total triiodothyronine dosage
  - 3 models were considered, Model 2 chosen because of normality in residuals, unrealistic TSH estimate at zero dose for Model 3.
- Number of doses/day had a positive coefficient of 1.09; each dose increase from 1-2-3 x day raises TSH by 1.09 units on average.

Dosings /Day	Predicted TSH (SE) at 100mcg T4
1	1.26 (0.35)
2	2.19 (0.29)
3	3.54 (0.57)
