# Supplementary material for: Clinical thyroidology: beyond the 1970s’ TSH-T4 Paradigm
Source: Front Endocrinol (Lausanne). 2025 Jun 24;16:1529791. doi: 10.3389/fendo.2025.1529791 (PMC12234311; doi:10.3389/fendo.2025.1529791)
Supplement: Supplementary file 2 [file SupplementaryFile2.pdf]

Supplementary Material

T4/T3 Combination Therapy Studies: Summary and Comment*

Smith 1970 (1)**: 1-for-1 oversubstitution in patients on clinically optimized T4 doses.** The TSH test was not yet available. Subjects were initially on clinically adjusted T4 doses of 200 or 300mcgs daily, much higher than today's TSH-normalizing doses. Each 100mcgs of the subject's T4 dose was replaced by a tablet containing 80mcgs of T4 and 20mcgs of T3. As oral T3 is generally around 3.3 times more potent than oral T4, this 1-for-1 oversubstitution produced a 50% increase in effective dose. For instance, subjects who had been considered clinically euthyroid on 300mcgs of levothyroxine were given 240mcgs of T4 and 60mcgs of T3. This marked overdosing produced negative effects in many subjects, as should have been expected. Even so, one-fifth preferred the combination, one-third of subjects preferred T4, and one-half had no preference.

Conclusion: Marked T3-for-T4 oversubstitution in patients who were on high, clinically optimized T4 doses. The authors admitted that their 1-for-1 substitution therapy had greater "biological effectiveness", yet still asserted that T4/T3 therapy had "shortcomings", that patients did not need T3, and that T4 monotherapy was superior. Their conclusions did not follow from their data. This study was cited by proponents of the TSH-T4 Paradigm as proof that the addition of T3 to T4 therapy was not indicated due to its "side effects" (2).

Cooke 1982 (3)**: T3 add-on study.** This was an open-label study of depressed patients who were on antidepressants and T4 monotherapy. On 100 to 500mcgs of T4 daily they all had TSH levels less than 1.0mIU/l. Several had low TSH levels. These subjects were on relatively high T4 doses compared to today's TSH-normalizing doses. 15 to 25mcgs of T3 were added to the subjects' T4 doses. This intervention resulted in a marked improvement in mood in 7 of 9 subjects, as judged by the clinicians and by rating scale scores. Most were still much improved 6 months later.

Conclusion: The addition of T3 to T4 therapy in depressed patients improved mood. The treatment TSH level was not measured, but would was probably undetectable with the addition of T3. This study suggests that the addition of a modest amount of T3 to T4 therapy is helpful even when the TSH is already low. It suggests that depression is related to inadequate T3 effect in the brain, and for some persons at least, T4 monotherapy does not restore euthyroidism in the brain

---

Bunevičius 1999 (4)**: TSH-maintaining equipotent 1-for-4 substitution study.** One-half of the subjects were receiving TSH-normalizing T4 therapy (TSHT4Rx) for Hashimoto's thyroiditis and the other half TSH-suppressive T4 therapy (TSHSupT4Rx) s/p thyroidectomy for thyroid cancer. 50mcgs of their T4 dose was replaced by 12.5mcgs T3, a nearly equipotent 1-for-4

* The studies are arranged by date, from the earliest to the latest.

substitution producing, on average, a 10:1 T4/T3 treatment ratio. This intervention normalized the average FT4 level which had been high. It raised the low-in-range average T3 to just mid-range at its near-peak level, 2 hrs after the daily dose. The average TSH decreased slightly from 0.8 to 0.5mIU/l. There were significant improvements in fatigue, mood, and some cognitive tests. Subjects preferred combination therapy by 10 to 1.

Conclusion: Equipotent T3-for-T4 substitution produced marked improvements at the same low-in-range TSH level. This study illustrates that the inclusion of T3 is helpful when TSH is low and particularly so in thyroidectomized patients on TSHSupRx. In them, the inclusion of T3 helps compensate for the loss of TSH-stimulated peripheral T4-to-T3 conversion and for the absence of the thyroid gland and its deiodinases.

Bunevičius 2000 (5)**: Reanalysis of the 1999 TSH-maintaining equipotent substitution study.** A subgroup of the previous study group (Bunevičius 1999) was analyzed, removing subjects with possible confounding features. The resulting group consisted of women only. Those on TSHT4Rx for Hashimoto's disease had lower FT4 and higher T3 values than did women on TSHSupRx post-thyroidectomy, as expected due to the presence or absence of the thyroid gland and its deiodinases. The thyroidectomy patients on T4, despite having 25% higher FT4 levels, had much lower T3 levels than the Hashimoto's patients, at the bottom of the reference range. On T4/T3 at the same TSH levels as on T4 therapy, both groups experienced significant improvements in mood and cognitive functioning. The improvements were more pronounced in the athyreotic women, as expected given their unphysiologically high FT4/T3 ratios and relative lack of T3. T4/T3 therapy produced less adverse effects than T4 monotherapy.

Conclusion: T4/T3 therapy was superior to T4 monotherapy at same low-in-range or low TSH levels, and was especially beneficial in athyreotic patients on TSHSupRx. The T4/T3 treatment ratio of 10:1 produced more pronounced improvements in athyreotic patients on TSHSupRx due to their reduced ability to convert T4 to T3.

Baisier 2001 (6): **Open-label, clinically optimized desiccated thyroid extract (DTE) treatment of patients with persisting symptoms on T4 monotherapy.** Three experienced endocrinologists who had always prescribed T4/T3 or DTE, noted that patients treated with TSHT4Rx had persisting symptoms of hypothyroidism and low 24-hour urine free T3 levels, similar to untreated hypothyroid patients (797.5pmol/24hrs, euthyroid mean 2000pmol/24hrs). They changed 40 patients to DTE, with gradually increasing doses. Their average T4 dose of 100mcgs/day was replaced by an average DTE dose of 233mgs/day (148mcgs T4 + 35mcgs T3). The TSH was not measured, but would have been low or undetectable in most. On clinically optimized DTE the patients' symptom scores improved markedly, by 70%. The 24hr. urinary free T3 rose to a near-normal 1990 pmol/L.

Conclusion: TSHT4Rx can leave patients highly symptomatic and leave urine free T3 levels in the hypothyroid range. DTE treatment, adjusted by clinical criteria, greatly improves hypothyroid symptoms and improves urine free T3 levels to those of euthyroid person. The authors' conclusion was that it is unethical to give patients TSHT4Rx.

Bunevičius 2002 (7)**: TSH-maintaining 1-for-5 undersubstitution study in thyroidectomized patients.** The subjects were women who had had a subtotal thyroidectomy for Graves' disease and were taking either 100 or 150mcgs of T4 to maintain low-in-range TSH levels. 50mcgs of their T4 dose was replaced by 10mcgs of T3: a 1-for-5 undersubstitution and therefore a reduction in effective dose. The resulting combination doses were either just 50mcgs of T4 and 10mcgs of T3 or 100mcgs of T4 and 10mcgs of T3. At 2 hrs. after the AM combination dose, at near-peak levels, T4/T3 combination therapy produced much lower FT4 levels and only slightly higher T3 levels than T4 monotherapy. TSH levels were similar—low-in-range in both groups (~0.46mIU/l). At the 24hr trough, T3 levels would have been lower on T4/T3 than on T4. The T4/T3 subjects therefore had lower average FT4 levels and lower average T3 levels. Yet, even with this undersubstitution subjects had less symptoms of both hypothyroidism and of hyperthyroidism and had improved mood. Subjects preferred T4/T3 by 3:1. Cardiac parameters revealed less thyroid effect upon the heart with combination therapy at the same TSH, indicating that the heart is especially sensitive to serum T4.

Conclusion: The inclusion of T3, even with 1-for-5 undersubstitution, was beneficial for most patients who had little-or-no residual thyroid tissue. In athyreotic patients, combination therapy produces benefits even with undersubstitution. The patients benefited despite much lower FT4 levels and similar FT3 levels. This suggests that T4-to-T3 conversion within tissues was improved with lower FT4 levels. The alleviation of both hypo- and hyperthyroid symptoms is consistent with the rat studies of Escobar-Morreale et al. On either T4 or T3 monotherapy some tissues will have excessive and others inadequate T3 effect. T4/T3 combination therapy in the right ratio for the patient works best to restore euthyroidism in all tissues.

Walsh 2003 (8)**: TSH-maintaining 1-for-5 undersubstitution study.** The subjects were mostly females with autoimmune hypothyroidism. They were receiving TSHT4Rx. 50mcgs of the their T4 dose (avg. 136mcgs) was replaced with 10mcgs of T3: another 1-for-5 undersubstitution. The average dose was just 76mcgs of T4 and 10mcgs of T3. With combined treatment, the 24-hr. trough TSH rose from 1.3 to 3.1mIU/l, the FT4 fell from 15.6 to 11.4pmol/l. The trough FT3 also fell from a low-in-range 3.7 to 3.5 (range: 3.0-5.5pmol/l). On average, there were no improvements in scales on T4/T3 and some deterioration in the general health questionnaire. On T4 therapy, the subjects had Zulewski scores in the hypothyroid range (3 or greater). The scores worsened on T4/T3, from 3.6 to 4.0. Cholesterol levels rose on combination therapy, indicating reduced T3 effect in the liver. Overall, subjects preferred T4 by a low ratio of 1.3-to-1. However, the subgroup whose TSH did not rise by more than 0.99mIU/l on T4/T3 did not differ in scales and had a slight preference for combination therapy.

Conclusion: 1-for-5 undersubstitution in patients with intact thyroid glands reduced overall T3 effect. Patients with intact glands have both residual T3 production and thyroidal deiodinases to increase T4-to-T3 conversion and add T3 to the blood that flows through the thyroid gland. They generally fare better on T4 monotherapy than athyreotic patients. The increases in anxiety and nausea on T4/T3 with a similar TSH were most likely due to hypocortisolism or increased inflammation in some of these autoimmune patients. Many T4/T3 studies used this inadequate 1-for-5 undersubstitution design—adding to the number of neutral and negative studies and vitiating meta-analyses of these studies.

Sawka 2003 (9)**: TSH-maintaining equipotent substitution study.** The subjects were almost all women on TSHT4Rx. They had intact thyroid glands and suffered from depression. Most probably had Hashimoto's thyroiditis. At baseline, the T4 group was relatively undertreated compared to the T4/T3 group (TSH 2.2mIU/l vs. 1.75mIU/l). Half of the subjects were given half of their usual T4 dose plus 12.5mcgs of T3 twice daily. The T3 dose was adjusted to maintain equal TSH concentrations in both groups. The mean T4 dose of 132mcgs/day was changed to 67mcgs of T4 and 19mcgs of T3 (an average 1-for-3.4 equipotent substitution). With study T4 treatment the TSH declined by 0.5mIU/l, while the T4/T3 group's TSH rose by 0.05mIU/l. There were improvements in both groups, but greater improvements in the T4/T3 group, especially in cognitive functioning, role-physical, role-emotional, mental health and social functioning scales. The subjects were not asked which treatment they preferred.

Comment: Equipotent 1-for-3.4 substitution produced improvements over T4 monotherapy, even at a higher TSH. The T4 group received a higher effective T4 dose with the study medication, helping to explain why both groups showed improvements. The study demonstrated that T4/T3 therapy is more effective than T4 monotherapy at the same mid-range TSH level, even in patients who likely have some thyroidal tissue. As is typical in such studies, the authors declared "no benefit" because the improvements did not reach an arbitrary standard for statistical significance. In considering all such studies, one must keep in mind that TSH-normalization with either T4 or T4/T3 is not clinically optimized treatment. Most studies indicate that it is undertreatment as expected due to the oversuppression of the TSH by once daily oral treatment. TSH-normalizing studies therefore are examining various degrees of undertreatment. All patients should have their T4, or better, T4/T3 doses adjusted according to clinical criteria, not the TSH.

Clyde 2003 (10)**: TSH-normalizing 1-for-3.3 equipotent substitution study.** Patients on T4 monotherapy, again mostly women with autoimmune thyroiditis, had an average TSH of 2.2mIU/l and an average FT4 of just 1.25ng/dl. The FT4 reference range was not stated; but was probably 0.8 to 1.8ng/dl. They were relatively undertreated at baseline compared to subjects in other studies who had low-in-range TSH levels and high-in-range FT4 levels. Their average T3 level at one hour after the T4/T3 dose was 135ng/dl (mid-range), indicating relative T4/T3 undertreatment. Both groups improved in hypothyroid quality-of-life (QOL) scores. The T4 group improved to a greater degree but had worse scores initially. Total and LDL cholesterol declined in T4/T3 group and increased in the T4 group.

Comment: 1-for-3.3 substitution was equally efficacious on average at the same mid-range TSH level in female ATD patients. Other studies found improvements on average in such patients with equipotent substitutions. However, this group is least likely to predictably improve with T3-for-T4 substitution. They have intact glands and therefore more T3-production than athyreotic patients. The TSH was not low or suppressed, so it was stimulating thyroidal and peripheral T4-to-T3 conversion. They also had a higher likelihood of hypocortisolism as they were mostly female, and inflammation as they had autoimmune thyroid disease. As discussed in the accompanying paper, thyroid replacement therapy (TRT) can worsen hypocortisolism and/or inflammation, and more effective T4/T3 combination therapy may worsen them more so, or possibly improve them in some patients by stimulating sufficient additional cortisol secretion. The inclusion of hypocortisol and/or inflamed patients could therefore worsen the average symptom and neuropsychological scores on combination therapy. This was clearly the case with

one female subject in this study. On T4/T3 therapy she had to drop out due to tremulousness, fatigue and inability to perform at work. Her TSH, FT4 and T3 were normal. She was not overdosed. She most likely suffered a worsening of relative hypocortisolism and/or inflammation.

Siegmund 2004 (11)**: TSH-reducing 1-for-1 oversubstitution study with low T3 doses to produce a 14:1 T4/T3 ratio.** The patients were again mostly women. Most of them had undergone thyroid removal/ablation. 9 of the 26 subjects had a history of autoimmune thyroid disease. 5% of subjects' TSH-normalizing T4 dose was substituted mcg-for-mcg with T3, a 1-for-1 oversubstitution—almost a T3 add-on study. For instance, for a person on 150mcgs of T4, the study dose was 142.5mcgs of T4 and 7.5mcgs of T3. The average initial TSH of 1.72mIU/l declined to 0.5mIU/l in the T4/T3 group. In 8 subjects on T4/T3 the TSH became undetectable, compared to only 2 subjects on T4. Mood was significantly impaired in 1/3rd of the subjects whose TSH was completely suppressed on combination therapy, yet their FT4 and FT3 levels were similar to the rest. Removing these subjects from the Beck Depression Inventory (BDI) scales revealed greater improvement in mood in the combination group. Even including those subjects, the BDI and some other subjective and objective scales improved nonsignificantly on T4/T3. One subject with a suppressed TSH on T4/T3 developed atrial fibrillation. Interestingly, the authors separately evaluated the subjects with autoimmune thyroid disease (ATD) and with non-autoimmune thyroid diseases (NTD). They noted that persons with ATD reported more psychopathological symptoms and negative mood states. They performed worse in neuropsychological tests on either regimen. These findings can be explained by hypocortisolism and/or inflammation—as expected with ATD. ATD subjects also had significantly higher FT3 levels, which is consistent with hypocortisolism as it increases T4-to-T3 conversion. Most of the subjects were women, and thus more prone to hypocortisolism. 6 of 26 subjects suffered from allergies and 3 had migraine headaches, again suggestive of relative hypocortisolism and/or inflammation. The authors blamed the negative symptoms on T4/T3 on "fluctuations in steady-state FT3 serum concentrations". However, persons without ATD reported improved mood and less anxiety on T4/T3.

Conclusion: The oversubstitution of T3 for T4 produced positive results in most patients, and negative results in others, especially those with ATD and a suppressed TSH. ATD patients are more likely to suffer from hypocortisolism and inflammation. The failure to achieve good results with T4/T3 therapy and/or the elicitation of hypocortisol symptoms should prompt an investigation of the patient's cortisol status and inflammatory conditions (e.g., autoimmune disease, chronic infection, etc.). Including some T3 is not a magic bullet. Some patients will not tolerate more T3 effect, they may not tolerate the T3 levels/effects of healthy controls. TRT must be individualized.

Escobar-Morreale 2005 (12)**: TSH-variable under- and oversubstitution study.** This was the only study that included a healthy control group for comparison. The subjects were 28 women with primary hypothyroidism, most with ATD. They were stable on T4 monotherapy and were randomly assigned to receive either 100mcgs of T4 or 75mcgs of T4 and 5mcgs of T3, a 1-for-5 undersubstitution. They were crossed over at 8 wks. Then in an 8-week T3 add-on period, all subjects received 87.5mcgs T4 + 7.5mcgs T3, a 1-for-1.67 oversubstitution. The average TSH levels were 1.95mIU/l on T4, 2.65mIU/l with undersubstitution and 1.09mIU/l with

oversubstitution therapy. With T3 oversubstitution 10 of the 28 women had low TSH levels. With undersubstitution, very little change was seen in any scales or metabolic parameters despite the higher TSH. Oversubstitution produced significant improvements in some cognitive tasks and in the visual analog scale for depression. Body temperature was slightly higher and LDL cholesterol lower with both T4/T3 regimens. Changing from T4 to oversubstitution produced increased depression and anger-hostility. These subjects would have had higher FT4 levels in the first few weeks due to T4's one week half-life, resulting in relative overtreatment during that time. All groups had lower quality-of-life scores than the healthy controls. Subjects on T4 had the highest average Zulewski score—in the hypothyroid range. On T4/T3 undersubstitution the Zulewski score decreased slightly. Only with T3 oversubstitution, with its frequent low TSH levels, was the Zulewski scale normalized—i.e., hypothyroid signs and symptoms were mostly eliminated. Interestingly, brainstem auditory evoked potential latencies were slowest in the T4 group, faster in the undersubstitution group and faster yet in the oversubstitution group, but still slower than in the controls. Likewise, ankle reflex relaxation time was lowest in the oversubstitution group, but still not as low as in controls. Subjects preferred the T4/T3 combinations over T4 by 9 to 1. Also, all treatment groups had worse scores on most scales and tests than did controls suggesting either that the T4/T3 doses were insufficient, of insufficient duration, and/or that many of these autoimmune patients had some degree of hypocortisolism.

Conclusion: T3 undersubstitution produced improvements in some hypothyroid-specific signs, symptoms and tests over T4 monotherapy. T3 oversubstitution produced greater improvements. On all three regimens patients remained hypothyroid compared to controls. The study found that in primary hypothyroidism, T3-for-T4 oversubstitution resulting in a lower or low TSH produced the most T3 effect but still left patients in a hypothyroid state. Many patients could have benefited from higher T4/T3 doses than supplied during oversubstitution. However, some did not tolerate higher T4/T3 levels/effects, most likely due to hypocortisolism and/or inflammation. There is no substitution for individualized, clinically optimized treatment.

Appelhoff 2005 (13)**: TSH-reducing study with fixed T4/T3 ratios.** The subjects were on TSHT4Rx for primary hypothyroidism due to ATD. They had low-in-range average TSH levels (~1.0mIU/l). The T4 dose was reduced and T3 added to maintain T4/T3 treatment ratios of 5:1 or 10:1. The median treatment TSH values in the T4, 10:1 and 5:1 groups were 0.64, 0.35, and 0.07mIU/l. Study medication was preferred to usual treatment by 29%, 41%, and 52% of the subjects, respectively. Despite randomization, the 10:1 and 5:1 groups had lower symptom scores at baseline, reducing the amount of benefit that could be seen. Objective testing revealed no differences except for a mean body weight change of +0.1, -0.5, and -1.7kgs, respectively. Those who preferred T4/T3 had lower TSH levels (0.07 to 0.35mIU/l) and lost more weight. No depressed subjects preferred T4, and all depressed subjects who received 5:1 treatment preferred it over T4.

Conclusion: T4/T3 combination therapy was superior at a TSH-similar 10:1 ratio, and more so at a TSH-suppressing 5:1 ratio. Patients with lower TSH levels experienced greater benefits. The inclusion of some males, who are less prone to hypocortisolism, probably reduced the incidence of adverse effects seen with T4/T3 therapy, highlighting the benefits of the lower T4/T3 ratio that produced low TSH levels.

Rodriguez 2005 (14)**: TSH-raising 1-for-5 undersubstitution study.** The subjects were on TSHT4Rx for primary hypothyroidism. The majority were female with ATD and intact thyroid glands. 50mcgs of each subject's T4 dose was replaced with 10mcgs of T3—a 1-for-5 undersubstitution and effective reduction in dose. T3 levels were higher in the substitution group because the blood was drawn after the morning dose. The avg. baseline TSH was 1.9mIU/l. The TSH levels increased in both groups—to 2.7mIU/l in the T4 group and 5.6mIU/l in the T4/T3 group. Most typical hypothyroid symptoms worsened with T3 undersubstitution yet these subjects lost 2.3kgs. Despite undersubstitution, subjects preferred T4/T3 therapy by 12 to 7.

Conclusion: The inclusion of T3 produced subjective benefits and weight loss even with undersubstitution that produced a high TSH and increased some hypothyroid symptoms. One can speculate that patients preferred combination therapy due to weight loss (not a trivial matter) or due to subtle mental/physical differences that could not be captured by the scales. Many of the combination studies found a tendency to weight loss with T3/T4 therapy, which shows that the inclusion of T3 improves energy production and/or utilization. This study indicates that the inclusion of some T3 is beneficial for most persons, even with relative undertreatment.

Saravanan 2005 (15)**: TSH-raising 1-for-5 undersubstitution/undertreatment study.** A large study (n=697) of patients with primary hypothyroidism on TSHT4Rx with a median TSH of 0.98mIU/l and average FT4 in the upper third of the reference range. They were on relatively higher T4 doses and had lower TSH levels than most patients. They were better treated than most. They were randomized to have 50mcgs of their T4 dose replaced by 10mcgs of T3; another 1-for-5 undersubstitution study with an effective reduction in dose. There was no crossover. The median TSH fell slightly to 0.75mIU/l in the T4 group but rose to 2.19mIU/l in the T4/T3 group, yet there were improvements in both groups in most symptom scales. The average FT4 levels were much lower in the T4/T3 group (1.15 vs 1.57ng/dl) and FT3 levels unchanged (low-in-range) at the 24hr. trough. The T4/T3 group had greater improvements in anxiety and other psychiatric measures at 3 months, especially those subjects with the highest trough FT3 levels, but the differences disappeared by 12 months.

Conclusion: Even with undersubstitution and higher TSH levels, T4/T3 combination therapy produced similar results to T4 monotherapy. Even though the TSH doubled in the T4/T3 group, there were no differences between the groups at 12 mos. This result, along with other studies, indicates that T4/T3 therapy produces more thyroid effect at any given normal TSH level, and even at somewhat higher TSH levels. The transient improvements in the T4/T3 group at 3 months were probably due to the higher FT4 levels in the first month or so after changing from T4 to T4/T3, due to T4's long half-life. These improvements were lost after FT4 levels fell to a lower steady state.

Regalbuto 2006 (16)**: Dose Adjusted to the same low TSH level with a fixed 3.5:1 T4/T3 ratio.** This was a crossover study involving patients, predominantly women, on TSHSupT4Rx after thyroidectomy for thyroid cancer. There were no controls for comparison. The doses in both arms were adjusted to keep the TSH around 0.2mIU/l. Combination therapy consisted of once-daily dosing with 3.5:1 T4/T3 tablets. The average T4 dose was 129mcgs/day, and T4/T3 dose was 97/28mcgs. T4/T3 therapy produced AM pre-dose trough FT4 levels that were slightly low, and FT3 levels that were low-in-range. No significant differences were seen in the subjects

during the two different regimens. Nearly half of subjects in both arms had intermediate Zulewski scores, i.e., had 3 to 5 significant hypothyroid signs or symptoms. No thyrotoxic symptoms were noted when T3 levels were superphysiological after the daily dose. Combination therapy produced small improvements in some measures of cognitive function. Equal numbers of subjects preferred T4 and T4/T3. Those who preferred T4 said that they felt slightly nervous on combined treatment, while those who preferred T3/T4 said that they felt more energetic and better overall.

Conclusion: T4/T3 therapy at a low 3.5:1 ratio was equivalent to T4 monotherapy at the same slightly low TSH levels in thyroidectomized cancer patients. T4/T3 produced no deleterious effects upon symptoms, heart rate, or blood pressure; or on cardiovascular, neurological or neuropsychological parameters. While no improvements were seen, this study demonstrated the safety and efficacy of T4/T3 therapy at a low ratio similar to DTE's 4:1 ratio. It also demonstrated that a low TSH on replacement therapy does not imply thyrotoxicosis. These were not symptomatic hypothyroid patients, and did not have autoimmune thyroid disease. They were asymptomatic and needed thyroidectomy for cancer. Therefore, they were more likely than symptomatic hypothyroid patients to have optimal TSH secretion, T4 to T3 conversion, and T3 sensitivity. Without ATD, they were less likely to have hypocortisolism. The low average FT4 and low-in-range FT3 levels at the 24hr trough 3.5:1 T4/T3 therapy were lower than what I typically see in persons on clinically optimized DTE therapy. Nearly half in both groups had intermediate Zulewski scores, so both groups were probably undertreated despite the low TSH. The optimal dose for any patient can only be determined by careful clinical evaluation including trials of higher or lower doses.

Slawik 2007 (17)**: 1-for-1 oversubstitution study in central hypothyroidism.** The patients were receiving T4-normalizing T4 therapy (1.1mcg/kg/d) that produced a mean 2hr. post-dose TSH that was normal (0.35mIU/l). This reflects clinicians' fear of a low TSH, even when they know that the patient has inadequate TSH production. A 70kg person received just 77mcgs of T4. These "empirically-treated" patients had below-mid-range FT4 and low-in-range FT3 levels—lower levels than most patients on TSHT4Rx for primary hypothyroidism. The subjects were given either a 45% higher T4 dose (1.6mcg/kg/d), or a 31% higher T4 dose (1.44mcg/kg/d) plus a T3 dose of 0.16mcg/kg/d. They were later crossed over. For a 70kg person the higher T4 dose was 112mcgs, and T4/T3 dose was 101mcgs of T4 and 11mcgs of T3—a 1:1 oversubstitution study. The high-dose T4 therapy and T4/T3 therapy both produced slightly high 2hr. peak FT4 levels. The T4/T3 therapy also produced high 2hr. peak FT3 levels, 50% higher than the upper limit of the reference range. In both groups the TSH was suppressed to <0.05mIU/l, as expected in the treatment of central hypothyroidism. Both the higher T4 and T4/T3 doses markedly reduced hypothyroid signs, symptoms and metabolic parameters, but T4/T3 combination therapy produced greater improvements. T4/T3 therapy lowered cholesterol and CPK levels, and improved muscle function and working memory to a greater degree than the higher T4 dose. Only T4/T3 therapy normalized the ankle reflex time. The average Zulewski score declined from 3.5 to 2.8 with the higher T4, dose, and to 2.6 on T4/T3. Only on T4/T3 were there no subjects with a Zulewski score >5. There were no signs or symptoms of excessive dosing on T4/T3 therapy. In fact, less adverse effects were reported in the T4/T3 group, despite their having high peak FT4 and FT3 levels.

Conclusion: In central hypothyroidism T4/T3 therapy at a higher effective dose was superior to a comparably higher T4 dose. The standard FT4-normalizing (often TSH-normalizing) therapy for central hypothyroidism was again shown to be inadequate. The TSH-T4 Paradigm is the cause of this common undertreatment in central hypothyroidism. These patients were helped by a higher T4 dose but were helped more by 10:1 T4/T3 combination therapy that produced slightly high FT4 and FT3 peak levels. The high FT3 peaks did not produce any negative symptoms or evidence of thyrotoxicosis, yet the authors feared that they signified thyrotoxicosis and so recommended against T4/T3 therapy. They dismissed the greater improvements on T4/T3 therapy and endorsed the higher T4 dose. Their conclusions were products of the TSH-T4 Paradigm, not of the evidence.

Nygaard 2009 (18)**: TSH-adjusted 1-for-2.5 oversubstitution study.** This was a crossover study of women with Hashimoto's thyroiditis who were receiving TSHT4Rx. They had low-normal TSH levels (avg. 1.1mIU/l). 50mcgs of their T4 dose was replaced by 20mcgs of T3, a 1-for-2.5 oversubstitution. The T4 dose was lowered to keep the TSH in both groups low in range (average 0.8mIU/l). The final average T4/T3 ratio was 4:1, identical to DTE. Significant improvements were seen in the T4/T3 group in 7 of 11 measures of quality of life, anxiety and depression. They had an average weight loss of 3.3 lbs. compared to none on T4. Subjects preferred combination therapy by 3 to 1. There were no differences in adverse effects between the treatment arms.

Conclusion: The inclusion of a larger 20mcg T3 dose in T4/T3 combination therapy produced marked benefits at the same low-in-range TSH levels in ATD patients. I believe that more benefit was seen with combination therapy than in most TSH-normalizing studies because the T3 dose was higher (20mcgs) and the overall T4/T3 ratio lower (4:1). In these ATD patients, the higher amount of T3 may have succeeded in stimulating sufficient additional cortisol secretion to both correct hypocortisolism and reduce inflammation. The T4 dose was adjusted to keep the TSH low-normal, guaranteeing moderate T4/T3 levels and T3 effects. The average daily dose was similar to 2grs (120mgs) of DTE.

Valizadeh 2009 (19)**: TSH-maintaining 1-for-4 equal substitution study.** The patients were mostly females with Hashimoto's thyroiditis. All were receiving TSHT4Rx (avg. TSH 2.3mIU/l). They were randomized to receive the same T4 dose or have 50mcgs of their T4 dose replaced with 6.25mcgs T3 twice daily (12.5mcgs)—a 1-for-4, nearly equal substitution. The T4 dose was adjusted as needed to maintain a normal TSH. There were no changes in any physical parameters at 4 months and no increase in adverse effects. In the T4/T3 group 3 of 4 GHQ-28 scores improved, whereas in the T4 group 2 of 4 scores worsened. The only statistically significant change was a reduction in anxiety/insomnia in the T4/T3 group. Subjects were not asked whether they preferred the study regimen or not.

Conclusion: T4/T3 therapy was superior as shown by improvements in the general health questionnaire. Only mild changes were noted, but they favored T4/T3. The improvements were dismissed by the authors as insignificant, but were most likely appreciated by the patients, and that is what matters in clinical medicine. Again, the authors conclude that T4 should remain the treatment of choice despite the improvements seen with T4/T3 therapy.

Hoang 2013 (20)**: TSH-adjusted DTE study.** This was the only randomized, double-blinded crossover study using DTE (Armour Thyroid®). The subjects were mostly female and were on TSHT4Rx for primary hypothyroidism. The average DTE dose was low, only 1.33grs (80mgs)—equivalent to 50mcgs T4 and 12mcgs T3. This was much lower than the average dose in Nygaard's positive study (77mcgs T4 + 20mcgs T3). The average TSH on DTE was also higher than in Nygaard: 1.67mIU/l vs. 0.8mIU/l. With T4 therapy, the RT3 level was high-in-range; with DTE it was reduced to mid-range. On DTE subjects lost an average of 3 lbs. Subjects preferred DTE by a ratio of 2.5 to 1. There were non-significant trends towards improved quality of life and better neuropsychological test scores in the DTE group, especially among those who preferred DTE. There were no adverse effects with either T4 or DTE.

Conclusion: At a slightly higher mid-range TSH level, DTE was superior to T4 monotherapy and produced no negative effects. This result is consistent with the other T4/T3 studies. DTE's 4:1 ratio was sufficient to lower the RT3 to mid-range. The lower RT3 along with the higher average FT3 produced greater T3 effect at a similar TSH level.

Pepper 2014 (21)**: Comparison of clinically adjusted T4 and DTE treatment.** This was an open label, clinical study. In the authors' practice, 1/3rd of a large group of patients with primary hypothyroidism had persisting symptoms of hypothyroidism on T4 therapy including fatigue, cold intolerance, constipation, myalgia, and unexplained weight gain. Their symptoms persisted despite clinical T4 dose adjustment that produced low TSH levels in many. They switched patients to DTE and again adjusted the dose to best eliminate hypothyroid symptoms, without regard for the TSH. The resultant average DTE dose was 92.3±28.6mgs (1.5±0.5grs), higher than Hoang's TSH-normalizing 80mg dose. The resultant average TSH was unchanged at 1.3mIU/l. However, around 50% of patients had low TSH levels on both therapies. Many patients who were still symptomatic on levothyroxine doses that produced low TSH levels obtained improvements on DTE doses that produced normal TSH levels. DTE therapy was rated as superior by 78% of patients. There were no significant adverse effects in either group. There were no correlations between satisfaction ratings and TSH levels or weight loss.

Conclusion: Clinically adjusted DTE is superior to clinically adjusted T4 monotherapy. Patients preferred DTE by a ratio of 4 to 1. This T4/T3 combination was superior at a similar average TSH level, often at a higher TSH level. A large percentage of patients had very low TSH levels (<0.01mIU/l) on clinically adjusted T4 and DTE, while some had high TSH levels. This emphasizes the uselessness of TSH levels for adjusting TRT. No untoward events occurred in the 30 patients who were over age 65, contradicting the unsubstantiated Beer's Criteria warning against DTE in seniors (22).

Kaminski 2016 (23): **Fixed-dose T4/T3 substitution in patients on different starting T4 doses, resulting in a 50-50 mixture of undersubstitution and equal substitution.** The subjects were mostly women with primary hypothyroidism. They were taking 125 or 150mcgs of T4. Their average TSH levels were low in the reference range. They were switched to 75mcgs of T4 and 15mcgs of T3 for 8 weeks. Given their different initial T4 doses, the result was a 50-50 mixture of 1-for-5 undersubstitution and 1-for-3.5 equal substitution—and therefore an overall reduction in overall dose for the group. The average TSH level was higher on T4/T3. On study T4 treatment, FT4 levels were higher and TSH levels lower, suggesting improved compliance or absorption. Both groups had improved symptom scores during the study, with non-significant

trends favoring T4/T3 for physical complaints and mood and emotion complaints. On T4/T3 FT4 levels were lower and trough FT3 levels unchanged. No adverse effects were reported. Subject preference was not assessed. There was no separate analysis of the subjects according to their different T4 doses prior to being switched to the fixed T4/T3 dose.

Conclusion: A mixture of 1-for-3.5 equal substitution and 1-for-5 undersubstitution showed some improvements in symptoms on T4/T3. The T4 arms also showed improvements due to higher effective dosing during the study.

Tariq 2018 (24): **Observational, retrospective study of TSH-normalizing, open label replacement of TSHT4Rx with DTE and 15:1 T4/T3 in patients with persisting hypothyroid symptoms.** Almost all subjects were women with primary hypothyroidism. The average DTE dose was only 30mgs (19mcgs T4 and 4.5mcgs T3), and average T4/T3 dose 75/5mcgs. Most of these subjects had residual thyroid function. The average TSH levels were normal and unchanged on DTE or T4/T3. Subjects reported feeling much better on both T4/T3 regimens and had good SF-20 scores. No scores were obtained prior to the change to T4/T3. No adverse effects were seen.

Conclusion: Substantial subjective benefits were obtained with TSH-normalizing DTE or T4/T3 treatment for patients with persisting hypothyroid symptoms on TSHT4Rx. Patients strongly preferred DTE and T4/T3 to T4. They preferred DTE at a slightly higher rate than T4/T3, despite the much lower T4/T3 dose with DTE. No adverse effects were seen. This suggests that DTE may contain T2 or other molecules that have some beneficial effects.

Heald 2021 (25): **Clinically adjusted open label replacement of TSHT4Rx with DTE in patients with persisting hypothyroid symptoms.** Almost all patients were women with ATD. The DTE dose ranged from 60 to 180mgs (avg. 123.5mgs), a higher dose than in Hoang, Tariq, and Shakir. The TSH on DTE ranged from <0.01 to 8.5mIU/L, therefore the DTE dose must have been adjusted clinically (though not stated by the authors). DTE produced large improvements in scores over the TSHT4Rx baseline. Those receiving >120mgs DTE had greater improvements in ThyPRO scores. Two patients experienced an increase in pre-existing palpitations.

Conclusion: Marked, often dramatic improvements in patient-reported well-being and in symptom scales with DTE adjusted by clinical criteria compared to TSHT4Rx. A few patients did experience an increase in palpitations that resolved with return to TSHT4Rx. Some persons cannot tolerate thyroid optimization therapy with T4 or T4/T3 due to hypocortisolism, inflammation, or cardiac problems.

Shakir 2021 (26): **TSH-normalizing controlled double blind study comparing T4 with DTE and 10:1 T4/T3.** Subjects were given low average doses: 115mcgs of T4, 77mgs of DTE, and 84mcgs of T4 with 9mcgs of T3. There was no clinical dose adjustment. Subjects were not selected by failure of TSHT4Rx, so were probably doing better on TSHT4Rx than in studies of dissatisfied patients. There were no differences in scales on DTE or T4/T3, except in the 1/3rd of subjects with the most symptoms at baseline. They improved significantly with DTE and T4/T3. More subjects favored DTE (45%) vs. T4/T3 (32%) and T4 (23%).

Conclusion: Both DTE and 10:1 T4/T3 were safe and were more effective for patients who had more hypothyroid symptoms on T4 monotherapy. Patients preferred DTE and T4/T3 overall. A hypothesis regarding the TSH-normalizing studies is that the high T3 peak levels after taking DTE and T4/T3 oversuppress the TSH @24hrs more than do the T4 peaks on T4 monotherapy, leading to relative underdosing with DTE and T4/T3. This would tend to limit the improvements on T4/T3 combinations to those persons with poor T4-to-T3 conversion.

References

1. Smith RN, Taylor SA, Massey JC 1970 Controlled clinical trial of combined triiodothyronine and thyroxine in the treatment of hypothyroidism. Br Med J 4:145-148. doi: 10.1136/bmj.4.5728.145
2. Evered D, Hall R 1972 Hypothyroidism. Br Med J 1:290-293. doi: 10.1136/bmj.1.5795.290
3. Cooke RG, Joffe RT, Levitt AJ 1992 T3 augmentation of antidepressant treatment in T4-replaced thyroid patients. J Clin Psychiatry 53:16-18. PMID: 1737734
4. Bunevicius R, Kazanavicius G, Zalinkevicius R, Prange AJ Jr 1999 Effects of thyroxine as compared with thyroxine plus triiodothyronine in patients with hypothyroidism. N Engl J Med 340:424-429. doi: 10.1056/NEJM199902113400603
5. Bunevicius R, Prange AJ 2000 Mental improvement after replacement therapy with thyroxine plus triiodothyronine: relationship to cause of hypothyroidism. Int J Neuropsychopharmacol 3:167-174. doi: 10.1017/S1461145700001826
6. Baisier WV, Hertoghe J, Eeckhaut W 2001 Thyroid insufficiency. Is thyroxine the only valuable drug? J Nutr Environ Med 11:159-166. https://doi.org/10.1080/13590840120083376
7. Bunevicius R, Jakuboniene N, Jurkevicius R, Cernicat J, Lasas L, Prange AJ Jr 2002 Thyroxine vs thyroxine plus triiodothyronine in treatment of hypothyroidism after thyroidectomy for Graves' disease. Endocrine 18:129-133. doi: 10.1385/ENDO:18:2:129
8. Walsh JP, Shiels L, Lim EM, Bhagat CI, Ward LC, Stuckey BG, Dhaliwal SS, Chew GT, Bhagat MC, Cussons AJ 2003 Combined thyroxine/liothyronine treatment does not improve well-being, quality of life, or cognitive function compared to thyroxine alone: a randomized controlled trial in patients with primary hypothyroidism. J Clin Endocrinol Metab 88:4543-4550. doi: 10.1210/jc.2003-030249
9. Sawka AM, Gerstein HC, Marriott MJ, MacQueen GM, Joffe RT 2003 Does a combination regimen of thyroxine (T4) and 3,5,3'-triiodothyronine improve depressive symptoms better than T4 alone in patients with hypo-thyroidism? Results of a double-blind, randomized, controlled trial. J Clin Endocrinol Metab 88:4551-4555. doi: 10.1210/jc.2003-030139
10. Clyde PW, Harari AE, Getka EJ, Shakir KM 2003 Combined levothyroxine plus liothyronine compared with levothyroxine alone in primary hypothyroidism: a randomized controlled trial. JAMA 290:2952-2958. doi: 10.1001/jama.290.22.2952
11. Siegmund W. Spieker K, Weike AI, Giessmann T, Modess C, Dabers T, Kirsch G, Sänger E, Engel G, Hamm AO, Nauck M, Meng W 2004 Replacement therapy with levothyroxine plus triiodothyronine (bioavailable molar ratio 14:1) is not superior to thyroxine alone to improve well-being and cognitive performance in hypothyroidism. Clin Endocrinol (Oxf) 60:750-757. doi: 10.1111/j.1365-2265.2004.02050.x
12. Escobar-Morreale HF, Botella-Carretero JI, Gómez-Bueno M, Galán JM, Barrios V, Sancho J 2005 Thyroid hormone replacement therapy in primary hypothyroidism: a randomized trial

comparing L-thyroxine plus liothyronine with L-thyroxine alone. Ann Intern Med 142:412-424. doi: 10.7326/0003-4819-142-6-200503150-00007
13. Appelhof BC, Fliers E, Wekking EM, Schene AH, Huyser J, Tijssen JG, Endert E, van Weert HC, Wiersinga WM 2005 Combined therapy with levothyroxine and liothyronine in two ratios, compared with levothyroxine monotherapy in primary hypothyroidism: a double-blind, randomized, controlled clinical trial. J Clin Endocrinol Metab 90:2666-2674. doi: 10.1210/jc.2004-2111
14. Rodriguez T, Lavis VR, Meininger JC, Kapadia AS, Stafford LF 2005 Substitution of liothyronine at a 1:5 ratio for a portion of levothyroxine: effect on fatigue, symptoms of depression, and working memory versus treatment with levothyroxine alone. Endocr Pract 11:223-233. doi: 10.4158/EP.11.4.223
15. Saravanan P, Simmons DJ, Greenwood R, Peters TJ, Dayan CM 2005 Partial substitution of thyroxine (T4) with tri-iodothyronine in patients on T4 replacement therapy: results of a large community-based randomized controlled trial. J Clin Endocrinol Metab 90:805-812. doi: 10.1210/jc.2004-1672
16. Regalbuto C, Maiorana R, Alagona C, Paola RD, Cianci M, Alagona G, Sapienza S, Squatrito S, Pezzino V 2007 Effects of either LT4 monotherapy or LT4/LT3 combined therapy in patients totally thyroidectomized for thyroid cancer. Thyroid 17:323-331. doi: 10.1089/thy.2006.0084
17. Slawik M, Klawitter B, Meiser E, Schories M, Zwermann O, Borm K, Peper M, Lubrich B, Hug MJ, Nauck M, Olschewski M, Beuschlein F, Reincke M 2007 Thyroid hormone replacement for central hypothyroidism: a randomized controlled trial comparing two doses of thyroxine (T4) with a combination of T4 and triiodothyronine. J Clin Endocrinol Metab 92:4115-4122. doi: 10.1210/jc.2007-0297
18. Nygaard B, Jensen EW, Kvetny J, Jarløv A, Faber J 2009 Effect of combination therapy with thyroxine (T4) and 3,5,3'-triiodothyronine versus T4 monotherapy in patients with hypothyroidism, a double-blind, randomised cross-over study. Eur J Endocrinol 161:895-902. doi: 10.1530/EJE-09-0542
19. Valizadeh M, Seyyed-Majidi MR, Hajibeigloo H, Momtazi S, Musavinasab N, Hayatbakhsh MR 2009 Efficacy of combined levothyroxine and liothyronine as compared with levothyroxine monotherapy in primary hypothyroidism: a randomized controlled trial. Endocr Res 34:80-89. doi: 10.1080/07435800903156340
20. Hoang TD, Olsen CH, Mai VQ, Clyde PW, Shakir MK 2013 Desiccated Thyroid Extract Compared With Levothyroxine in the Treatment of Hypothyroidism: A Randomized, Double-Blind, Crossover Study. J Clin Endocrinol Metab 98:1982-1990. doi: 10.1210/jc.2012-4107
21. Pepper GM, Casanova-Romero PY 2014 Conversion to Armour Thyroid from levothyroxine improved patient satisfaction in the treatment of hypothyroidism. J Endocrinol Diabetes Obes 2:1055-1059. doi: 10.47739/2333-6692/105
22. The American Geriatrics Society 2012 Updated Beers Criteria for potentially inappropriate medication use in older adults. J Am Geriatr Soc 60:616-631 (Table 2). doi: 10.1111/j.1532-5415.2012.03923.x
23. Kaminski J, Miasaki FY, Paz-Filho G, Graf H, Carvalho GA. Treatment of hypothyroidism with levothyroxine plus liothyronine: a randomized, double-blind, crossover study. Arch Endocrinol Metab. 2016 Nov-Dec;60(6):562-572. doi: 10.1590/2359-3997000000192
24. Tariq A, Wert Y, Cheriyath P, Joshi R. Effects of Long-Term Combination LT4 and LT3 Therapy for Improving Hypothyroidism and Overall Quality of Life. South Med J. 2018 Jun;111(6):363-369. doi: 10.14423/SMJ.0000000000000823

25. Heald AH, Premawardhana L, Taylor P, Okosieme O, Bangi T, Devine H, Livingston M, Javed A, Moreno GYC, Watt T, Stedman M, Dayan C, Hughes DA. Is there a role for natural desiccated thyroid in the treatment of levothyroxine unresponsive hypothyroidism? Results from a consecutive case series. Int J Clin Pract. 2021 Dec;75(12):e14967. doi: 10.1111/ijcp.14967
26. Shakir MKM, Brooks DI, McAninch EA, Fonseca TL, Mai VQ, Bianco AC, Hoang TD. Comparative Effectiveness of Levothyroxine, Desiccated Thyroid Extract, and Levothyroxine+Liothyronine in Hypothyroidism. J Clin Endocrinol Metab. 2021 Oct 21;106(11):e4400-e4413. doi: 10.1210/clinem/dgab478
